# *In vitro* sulfuration of *Rhodobacter capsulatus* formate dehydrogenase

**DOI:** 10.1016/j.jbc.2025.108511

**Published:** 2025-04-15

**Authors:** Benjamin R. Duffus, Benedict J. Elvers, Christian Teutloff, Carola Schulzke, Silke Leimkühler

**Affiliations:** 1 Institute of Biochemistry and Biology, Department of Molecular Enzymology, University of Potsdam, Potsdam, Germany; 2 Institute of Biochemistry, Department of Bioinorganic Chemistry, University of Greifswald, Greifswald, Germany; 3 Institute of Experimental Physics, EPR Spectroscopy of Biological Systems, Freie Universität Berlin, Berlin, Germany

**Keywords:** molybdenum, tungsten, formate, carbon dioxide, iron-sulfur protein, sulfur, sulfur transfer, dithionite, hydrogen sulfide

## Abstract

Metal-dependent formate dehydrogenases (FDHs) are of considerable interest as a bioinspired metalloenzyme target to efficiently reduce the greenhouse gas CO_2_ into the portable energy carrier formate under physiological conditions. These enzymes were shown to harbor an active site sulfido ligand that is essential for the formate oxidation and CO_2_ reduction activity and contributes to the oxygen sensitivity of the enzyme, since the ligand is rapidly lost in the presence of O_2_. Inhibitors like azide or nitrate are routinely employed to protect the active site from oxidative damage. The demonstrated unitary *in vitro* sulfido ligand incorporation to the active site bis metal-binding pterin guanine dinucleotide (bis-MGD) cofactor in FDH from *Rhodobacter capsulatus* of this study also completely reactivates the enzyme. Reductive treatment with either sulfide or bisulfite, or with sodium dithionite under weakly acidic conditions in the strict absence of O_2_ resulted in comparable enzymatic activity to FDH purified after heterologous expression in *Escherichia coli*. Confirmation of the inserted sulfido ligand was afforded by EPR spectroscopy of a Mo^V^ intermediate species associated with MoS_6_ coordination. Specific insertion of a ^33^S sulfido ligand to the bis-MGD Mo evidenced the chemical insertion of the sulfido ligand and confirmed its role to serve in defining the electronic character of the sulfurated bis-MGD Mo^V^-SH state. The relevance of these results, in relation to known *in vitro* sulfuration assays described for other molybdoenzymes, is discussed.

Molybdenum and tungsten are important and essential trace elements in biological systems that serve in key steps of carbon, sulfur, and nitrogen metabolism (1, 2). Apart from the unrelated FeMoCo nitrogenases, three distinct classes of molybdoenzymes and tungstoenzymes exist, which qualitatively differ in their coordination of ligands to the molybdenum ion. These classes are known as the xanthine oxidase/dehydrogenase (XO/XDH), the sulfite oxidase (SO), and the dimethylsulfoxide (DMSO) reductase families. One characteristic of the XO/XDH family is a Mo-bound sulfido ligand that is catalytically essential for the hydride transfer step of its reaction (3), as was shown for bovine milk XO (4) and chicken liver XDH (5). The sulfide was unambiguously shown to be one of the ligands of molybdenum (Mo=S) by EPR spectroscopy (6, 7). Previously, unusual CN^–^ inhibition was identified for XO (8) where the ion bound to the sulfido ligand, resulting in the release of SCN^-^ and the formation of an inactive form (desulfo form); this reaction is present also naturally in a significant amount in eukaryotes (4). Later, it was revealed that a chemical reconstitution of the sulfido ligand is possible, by incubation of the enzyme with sulfide and/or dithionite under reducing conditions, recovering almost full activity (9).

Metal-dependent formate dehydrogenases (FDHs) represent a group of diverse enzymes in bacteria and archaea, which catalyze the reversible two electron reduction of CO_2_. The active site comprises a molybdenum or tungsten metal ion binding two molybdopterin guanine dinucleotides (bis-MGD), a protein-based Cys or Sec residue, and a participatory sulfido ligand (Fig. 1). Their sulfido ligand, similar to XO/XDH, is involved in a hydride abstraction to support the oxygen atom transfer reaction of formate dehydrogenases and likely is important for other members of the DMSO reductase family as well. Our proposed OAT reduction of CO_2_, comprising first the conversion of the substrate to carbonic acid/bicarbonate and then the extrusion of an oxygen atom from the substrate's C-O-H group to yield water plus formate as a potentially industrial useful reduced carbon oxide form (6, 10, 11, 12, 13), is totalized as follows:

(1) HCO_3_^–^ + 2 e^–^ + 2 H^+^ ⇌ HCO_2_^–^ + H_2_O

**Figure 1:**
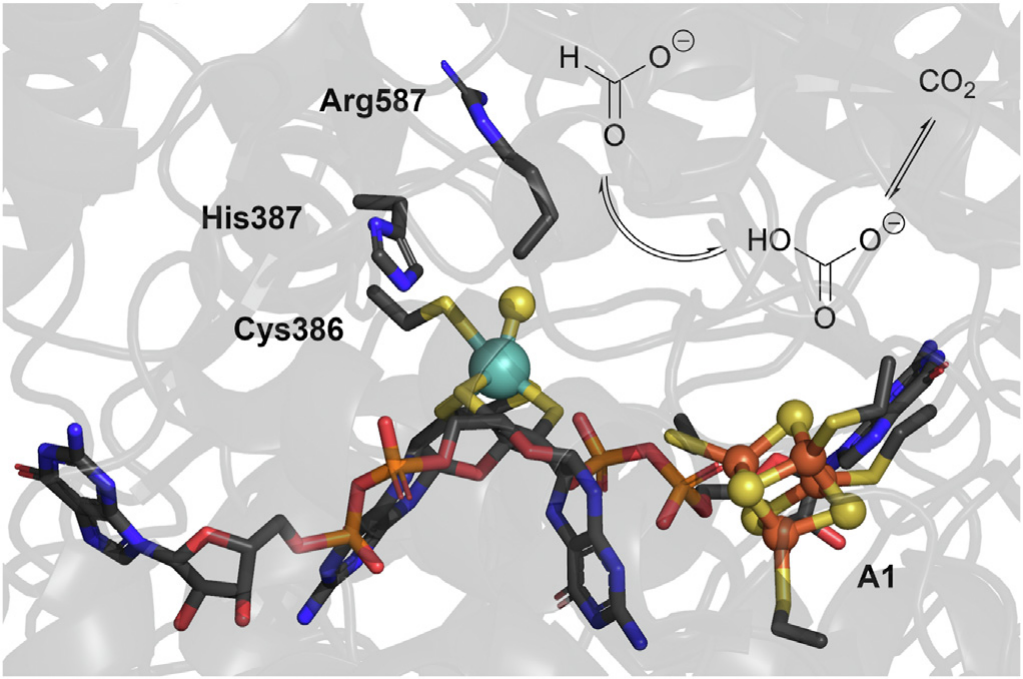
**The active site of *Rhodobacter capsulatus* FDH, depicting the bis-MGD cofactor and its surroundings.** Key active site residues in proximity to the active site, along with the A1 [4Fe-4S] cluster and the reaction catalyzed are depicted. The Mo, Fe, as well as the sulfido components for the bis-MGD cofactor and [4Fe-4S] cluster respectively are depicted as spheres, while other emphasized components are depicted in the stick structure. The structure represents the as-isolated EM map of FDH (PDB entry 6TGA). The image was prepared in PYMOL 2.5.7.

In FDH enzymes, the sulfido ligand mainly contributes to their oxygen sensitivity, since it is lost upon exposure to oxygen, resulting in an inactive enzyme with an oxo ligand present instead of a sulfido ligand (14). In the presence of the inhibitor azide, *Rhodobacter capsulatus* FDH can be isolated with a quantitatively bound sulfido ligand following aerobic purification (15). The inhibitor azide stabilizes the sulfido ligand, suggesting that it binds in vicinity of the sulfido ligand and prevents contact of the active site sulfide with O_2_ (16). Functionally, the coordination of the active site sulfido ligand *versus*, for example, a non-native oxido ligand further impacts the redox potential of the central Mo or W metal ion, which may bias the catalytic reaction (17, 18). Consequently, loss or lack of insertion of the sulfido ligand at the active site Mo or W ion typically renders the active site catalytically incompetent, most significantly, with regard to hydride transfer as an inherent part of the enzymatic cycle (4, 18, 19, 20).

The biosynthetic insertion of the sulfido ligand in mononuclear molybdoenzyme and tungstoenzyme involves a dedicated molecular chaperone and an l-cysteine desulfurase, which links the MPT and the iron-sulfur (Fe-S) cluster maturation pathways (21, 22). Similarly, in *Escherichia coli* FDHs, the sulfur is primarily incorporated into MGD by an MGD-binding chaperone-like FdhD together with the housekeeping l-cysteine desulfurase IscS. This emergent view regarding sulfuration of the bis-MGD cofactor also for FDH enzymes is supported by the crystal structure of *E. coli* FdhD, which has provided insights regarding sulfuration *in vivo* (23). Isolated as a homodimeric complex in the presence of GDP, a surrogate of the bis-MGD cofactor, the dimer forms a tunnel, in which the bis-MGD cofactor is able to bind and by which IscS is aligned so that it can form a favorable interaction. As was noted previously, the identified CXXC motif forms a structurally disordered loop region on one end of the dimer tunnel that can interact at the interface and support IscS binding (23, 24, 25).

So far, successful reinsertion of the sulfido ligand in FDH by chemical methods as comparable to biosynthetic methods has not been demonstrated. Inspired by the similarities in behavior and interaction between the cognate chaperone proteins FdhD in *E. coli* and FdsC in *R. capsulatus* with *E. coli* IscS, the *in vitro* sulfuration of *R. capsulatus* FDH was investigated in detail. Unitary *in vitro* sulfido ligand incorporation in FDH was observed in a catalytically incompetent form of the enzyme coordinating the bis-MGD cofactor by incubating it with the sulfur source sodium sulfide in the presence of a reducing agent that was comparably active to FDH expressed in the presence of the maturases FdsC and FdhD. Sulfide or (bi)sulfite in the presence of reductant served as competent sulfur sources resulting in substantial enzymatic formate oxidation activity; the latter also scavenged any present O_2_ which would impede *in vitro* sulfuration. In turn, *in vitro* chemical sulfido ligand addition to the bis-MGD cofactor is generally and uniformly possible without the requirement of the sulfuration-associated chaperone protein, an aspect that might be of great advantage to reactivate oxidatively damaged FDH enzymes in assays for formate production applications.

## Results

### Cyanolysis and inactivation of *Rc*FDH^WT^

To test whether the sulfido ligand in FDH could be removed with cyanide and released as thiocyanate resulting in the inactivation of the enzyme, as being characteristic for enzymes of the XO family (4, 9, 26, 27, 28, 29), *R. capsulatus* FDH^WT^ was incubated with cyanide under anaerobic conditions. Treatment of 30 *μ*M *Rc*FDH^WT^ with 1 mM KCN for 30 min at 10 °C resulted in a complete loss of formate oxidation activity with methyl viologen as an electron acceptor, as compared to 7040 ± 320 min^-1^ activity for the aerobically purified enzyme (Fig. S1). A similar result was observed with NAD^+^ as an electron acceptor. The released thiocyanate quantity was detected to be 10 ± 1 *μ*M (Fig. S1). This is roughly comparable to the concentration of molybdenum, [Mo], of approximately 12.6 *μ*M and is consistent with the sulfido ligand loading (76.3%) on *Rc*FDH^WT^, accompanying aerobic purification in the presence of azide (15). The value confirms the essentially quantitative loss of the sulfido ligand and its release as thiocyanate upon cyanide treatment and shows that the sulfido ligand of FDH is also cyanolyzable.

### *In vitro* sulfuration of *Rc*FDH^ΔFdsC^

**Figure 2:**
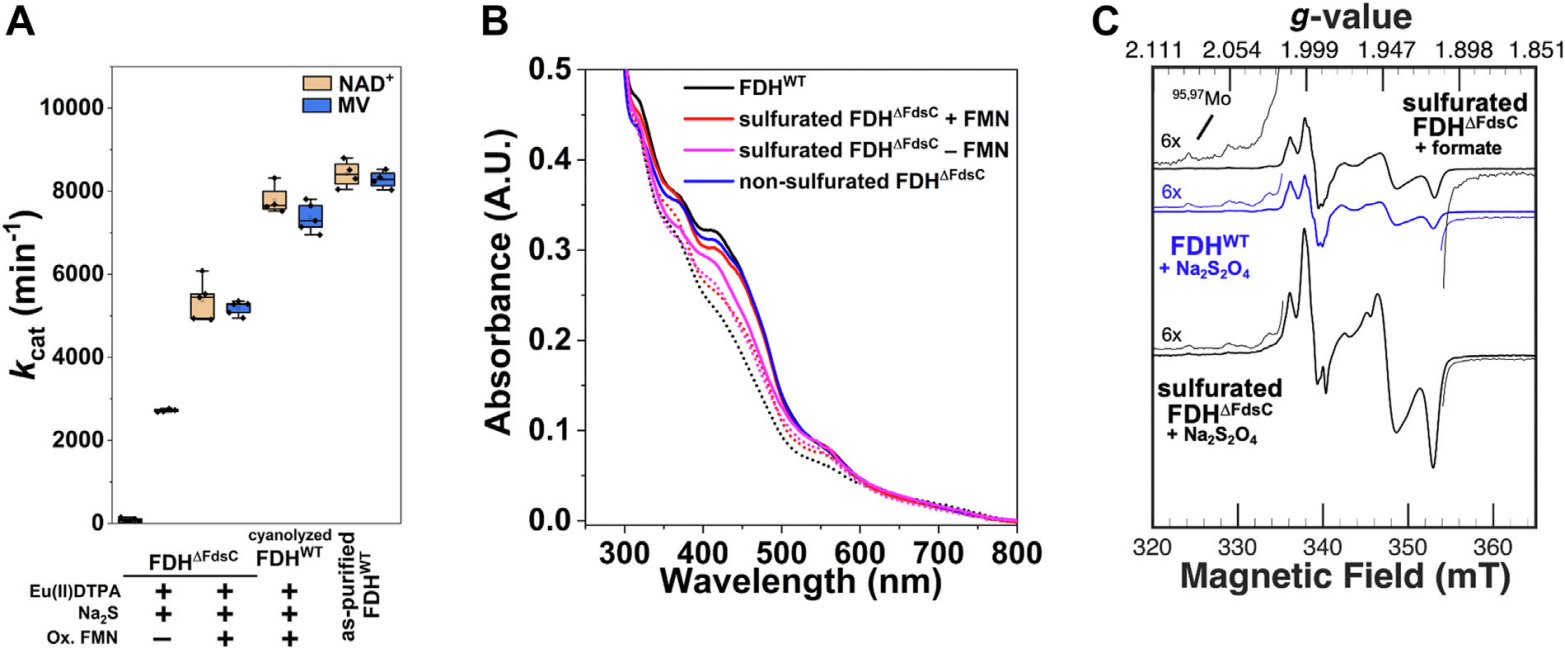
**Reductant and sulfide-dependent bis-MGD sulfuration.***A*, depicts formate oxidation activities of the sulfurated *Rc*FDH^ΔFdsC^ or cyanolyzed *Rc*FDH^WT^ following *in vitro* sulfuration with Na_2_S in the presence of Eu^II^DTPA and NaN_3_. For *Rc*FDH^ΔFdsC^, the absence and presence of oxidative FMN reconstitution performed after sulfuration is depicted. Shown activity measurements represent formate oxidation using either NAD^+^ or methyl viologen (MV) as an electron acceptor. As an activity reference, as-isolated, azide-inhibited (pre-sulfurated) *Rc*FDH^WT^ is depicted. Please see the Experimental procedures section “Activity measurements” for more information regarding the formate oxidation activity assay applied here. *B*, depicts UV-Vis reduction spectra of sulfurated *Rc*FDH^ΔFdsC^ upon treatment with formate. *Solid* lines depict FDH samples in the as-isolated or as-obtained state following *in vitro* sulfuration, while the *dotted* lines depict respective spectra reduced with 5 mM sodium formate. For spectral comparison, reduction of sulfurated *Rc*FDH^WT^ with formate and the spectrum of as-isolated, nonsulfurated *Rc*FDH^ΔFdsC^ are depicted as reference spectra, with respective spectra normalized relative to the FMN-associated and Fe-S cluster absorbances at 478 nm and 550 nm. *C*, depicts CW X-band EPR spectra of sulfurated *Rc*FDH^ΔFdsC^ following *in vitro* sulfuration with Na_2_S in the presence of Eu^II^DTPA without oxidative FMN treatment. Sulfurated samples (*black traces*) were reduced with either 10 mM formate or 10 mM Na_2_S_2_O_4_ in 75 mM potassium phosphate, 10 mM NaN_3_, pH 7.5. The *blue* trace depicts *Rc*FDH^WT^ reduced with 10 mM formate in the same buffer as a reference spectrum. Spectral data was obtained at a microwave frequency of 9.43 GHz at 110 K with a 2 G modulation amplitude at 4 mW microwave power and at 100 kHz modulation frequency. The sulfurated *Rc*FDH^ΔFdsC^ spectra represent a [FDH] of 265 μM; the *Rc*FDH^WT^ spectrum was normalized relative to the Mo^V^ species present in formate-reduced, sulfurated *Rc*FDH^ΔFdsC^. For spectral deconvolution of the Na_2_S_2_O_4_-reduced, sulfurated *Rc*FDH^ΔFdsC^ spectrum, see Fig. S2.

**Figure 3:**
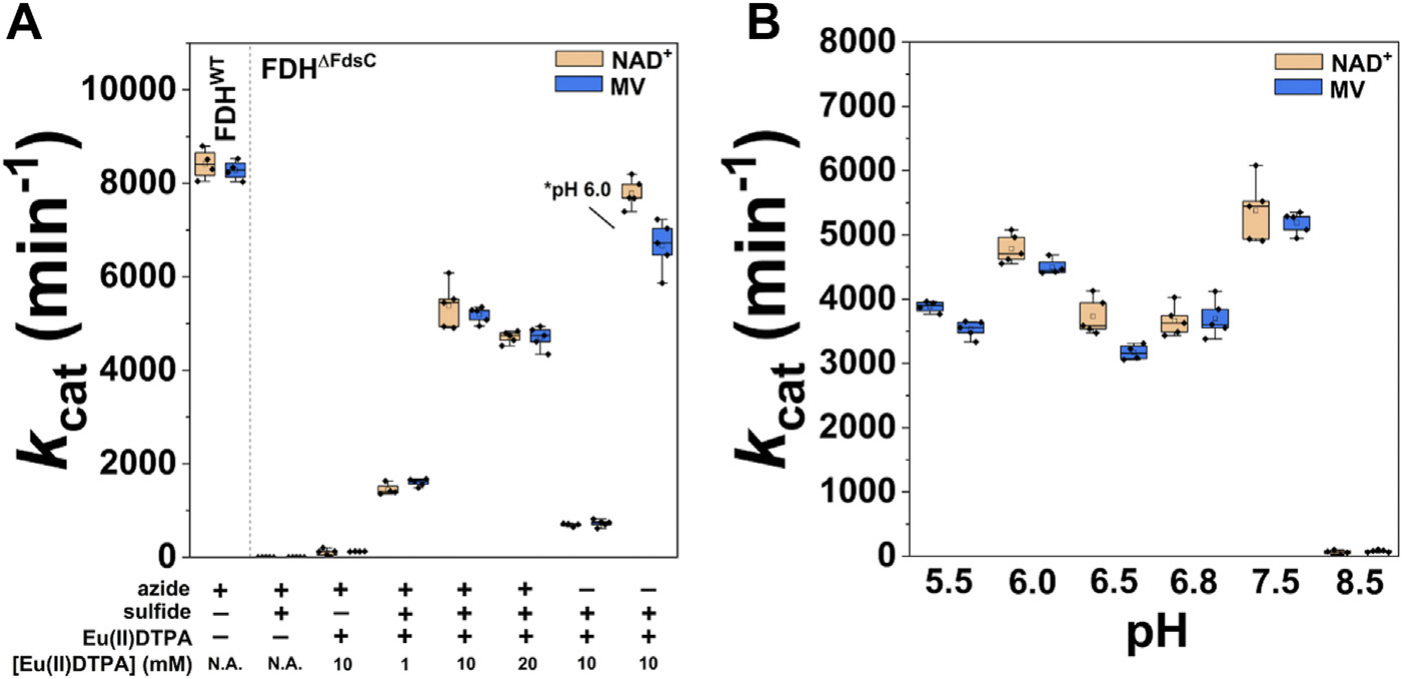
**Assessment of optimization of *in vitro* sulfuration of *Rc*FDH^ΔFdsC^.***A*, depicts conditions by which *Rc*FDH^ΔFdsC^ was sulfurated in 75 mM potassium phosphate, pH 7.5 in the presence or absence of 10 mM NaN_3_. Variation relative to the initially defined sulfuration conditions (2.5 h incubation with Eu^II^DTPA and 10 mM Na_2_S) is shown. The condition-optimized sulfuration depicted on the far *right* of the panel was performed in 75 mM potassium phosphate, pH 6.0 buffer. *B*, represents defined sulfuration components (10 mM Na_2_S and 10 mM Eu^II^DTPA) but with altered pH of the azide-containing sulfuration buffer. In all cases, activity was measured in 100 mM Tris–HCl, pH 9.0 in an anaerobic Coy chamber, with 6 mM formate, and either 100 *μ*M methyl viologen or 2 mM NAD^+^ at ambient temperature. Measurements represent at least five measurements. N.A. = not added.

After the successful inactivation of FDH with cyanide, we wanted to determine whether a chemical resulfuration is also possible for this enzyme, since unlike XO/XDH, FDH belongs to the DMSO reductase family. So far, studies directed toward the *in vitro* sulfuration have been limited (24) or unsuccessful (4, 18, 24). To avoid other potential damage to the enzyme caused by the cyanide treatment, we used the completely inactive 100% oxo-containing *Rc*FDH^ΔFdsC^ (heterologously expressed *R. capsulatus* FDH in *E. coli* lacking both FdhD and FdsC) and performed a simple incubation with sodium sulfide under anaerobic conditions. However, these first trials were unsuccessful, implicating the need for additional components in the sulfuration assay. Since sulfuration of molybdoenzymes such as XDH are also known to require the presence of a reductant (9), reduction in the presence of sulfide and different reducing agents were tested. The reducing agent Eu^II^diethylenetriaminepentaacetic acid (DTPA) (30) was first selected as a sulfur-free, low-potential reductant that is not expected to directly interact with the bis-MGD cofactor. Following incubation of *Rc*FDH^ΔFdsC^ with 10 mM Eu^II^DTPA, 10 mM Na_2_S, and 10 mM NaN_3_ at pH 7.5 for 2.5 h, apparent formate oxidation activity using oxidized methyl viologen as an electron acceptor could be observed (Fig. 2*A*). However, no formate oxidation activity was observed in parallel measurements with NAD^+^ as an electron acceptor, suggesting redox-affected changes at the diaphorase subunit that binds the riboflavin 5′-monophosphate (FMN) cofactor. Since these results implicated loss of the FMN cofactor due to reductive treatment, FMN cofactor reconstitution under oxidative conditions was performed following sulfuration. This step resulted indeed in concomitant formate oxidation activity with either NAD^+^ or oxidized methyl viologen as electron acceptors (Fig. 2*A*). The level of formate-based reduction determined for the chemically sulfurated/FMN-reconstituted sample *via* its UV-Vis absorbance spectrum was similar to that of natively sulfurated *Rc*FDH^WT^ (Fig. 2*B*, see also Fig. S2). An interesting issue in this regard is the question whether to include azide in the sulfuration experiments or not. Initially, omitting azide during reductive incubation with sulfide resulted in apparent formate oxidation activity substantially lowered to 25 to 50% relative to that in the presence of azide (see Fig. 3). However, as will be described in more detail below, performing the sulfuration at pH 6.0 in the absence of azide with freshly degassed sulfuration buffer under strictly anaerobic conditions yielded reliably comparable sulfuration levels to what could be achieved at pH 7.5 in the presence of azide.

We also tested the cyanide-inactivated *Rc*FDH^WT^ for *in vitro* resulfuration by incubating it with 10 mM Na_2_S for 2.5 h in the presence of 10 mM Eu^II^DTPA. Following the workup procedure described for *Rc*FDH^ΔFdsC^, resulfurated *Rc*FDH^WT^ exhibited formate oxidation activity of 5700 ± 220 min^−1^ and 4340 ± 480 min^−1^ with methyl viologen and NAD ^+^ as electron acceptors (Fig. 2*A*, see also Fig. S1), which is comparable to those observed with sulfurated *Rc*FDH^ΔFdsC^.

To confirm the apparent sulfido ligand insertion by chemical sulfuration into *Rc*FDH^ΔFdsC^, EPR spectroscopy was performed (Fig. 2*C*). The sample, prepared as described above, upon reduction with Na_2_S_2_O_4_ gave a composite EPR signal of different overlapping *S =* 1/2 Mo^V^ species (see Fig. S3 for a composite simulation of the particular species contributing to the spectrum). The mixture of Mo^V^ signals could be assigned to a Mo^V^ species associated with nonsulfurated, Na_2_S_2_O_4_-reduced *Rc*FDH^ΔFdsC^ (with *g-*values of 2.010, 1.986, and 1.954 – see Table S1 and Figs. S4 and S5) and to the sulfurated cofactor, similar to reduced *Rc*FDH^WT^ enzyme published previously (Fig. 2*C*, see also Figs. S3 and S4) (31, 32). By spectral simulation, a ratio of the sulfurated and nonsulfurated Mo^V^ species was estimated to 50:50 (Fig. S3). By comparison, reduction of the same sulfurated enzyme stock with 10 mM sodium formate under the same conditions resulted in the sulfurated, azide-inhibited Mo^V^ signal (Fig. 2*C*, see also Fig. S6 and Table S1) (32), which demonstrated that *in vitro* sulfuration was incomplete.

### Optimization of *in vitro* sulfuration

After the apparent *in vitro* insertion of the sulfido ligand was confirmed, optimal conditions for *in vitro* sulfuration of *Rc*FDH^ΔFdsC^ with sulfide were assessed (Fig. 3). Incubation in the presence of Eu^II^DTPA and the absence of sulfide resulted in a reproducible, but low observed activity. This is likely either due to the presence of endogenously bound Fe-S clusters or due to the small amount of apo-enzyme lacking the bis-MGD cofactor that potentially has a low amount of labile sulfide due to the substantial number of Fe-S clusters bound in *Rc*FDH (Fig. 3*A*). By comparison, incubation of *Rc*FDH^ΔFdsC^ with 10 mM Eu^II^DTPA in the absence of azide resulted in no apparent formate oxidation activity (data not shown). A substantial increase in activity was observed by increasing the assay sulfide concentration, with 10 mM Na_2_S in 75 mM potassium phosphate, 10 mM NaN_3_, pH 7.5 that gave reproducible positive results. However, simultaneously decreasing the amounts of FDH, sulfide, and Eu^II^DTPA while maintaining their ratios resulted in less apparent sulfuration. Next, the impact of the [Eu^II^DTPA] was assessed. A 10 mM Eu^II^DTPA assay concentration was found to be optimal, with less FDH becoming precipitated upon incubation, while ensuring sufficient reductant excess. A 20 mM Eu^II^DTPA assay concentration resulted in a slightly decreased activity at the expense of increased precipitation, while lowering the Eu^II^DTPA concentration to 1 mM resulted in approximately one third the activity observed relative to 10 mM at pH 7.5 in the presence of azide, reflecting the need for a reducing agent (Fig. 3*A*). Then the pH value for the sulfuration with sulfide/Eu^II^DTPA in the presence of azide was optimized. While substantial sulfuration was observed below pH 7.5, at pH 7.5, an improved apparent sulfuration was achieved (Fig. 3*B*). By comparison, almost no sulfuration was observed at pH 8.5. As noted above, these sulfuration conditions appeared to require the presence of azide to sulfurate the bis-MGD cofactor. Since substantial sulfuration could be observed at acidic pH values (approximating near the p*K’*_a_ of H_2_S) (33), sulfuration at pH 6.0 was attempted again albeit in the absence of azide. When performed using freshly degassed sulfuration buffer, comparable formate oxidation rates could be attained relative to as-purified, sulfurated *Rc*FDH^WT^ enzyme (Fig. 3*A*), although the enzyme was less stable at this pH, due to a similar pI value (5.99) for the enzyme. Similar results were achieved with cyanolyzed *Rc*FDH^WT^ upon sulfuration at pH 6.0 in the absence of azide using freshly degassed sulfuration buffer (Fig. S1). Thus, the optimal conditions for *in vitro* sulfuration with sulfide are as follows: 10 mM Eu^II^DTPA and 10 mM Na_2_S at pH 6.0, in the absence of azide.

### *In vitro* sulfuration of *Rc*FDH^ΔFdsC^ in the presence of dithionite

**Figure 4:**
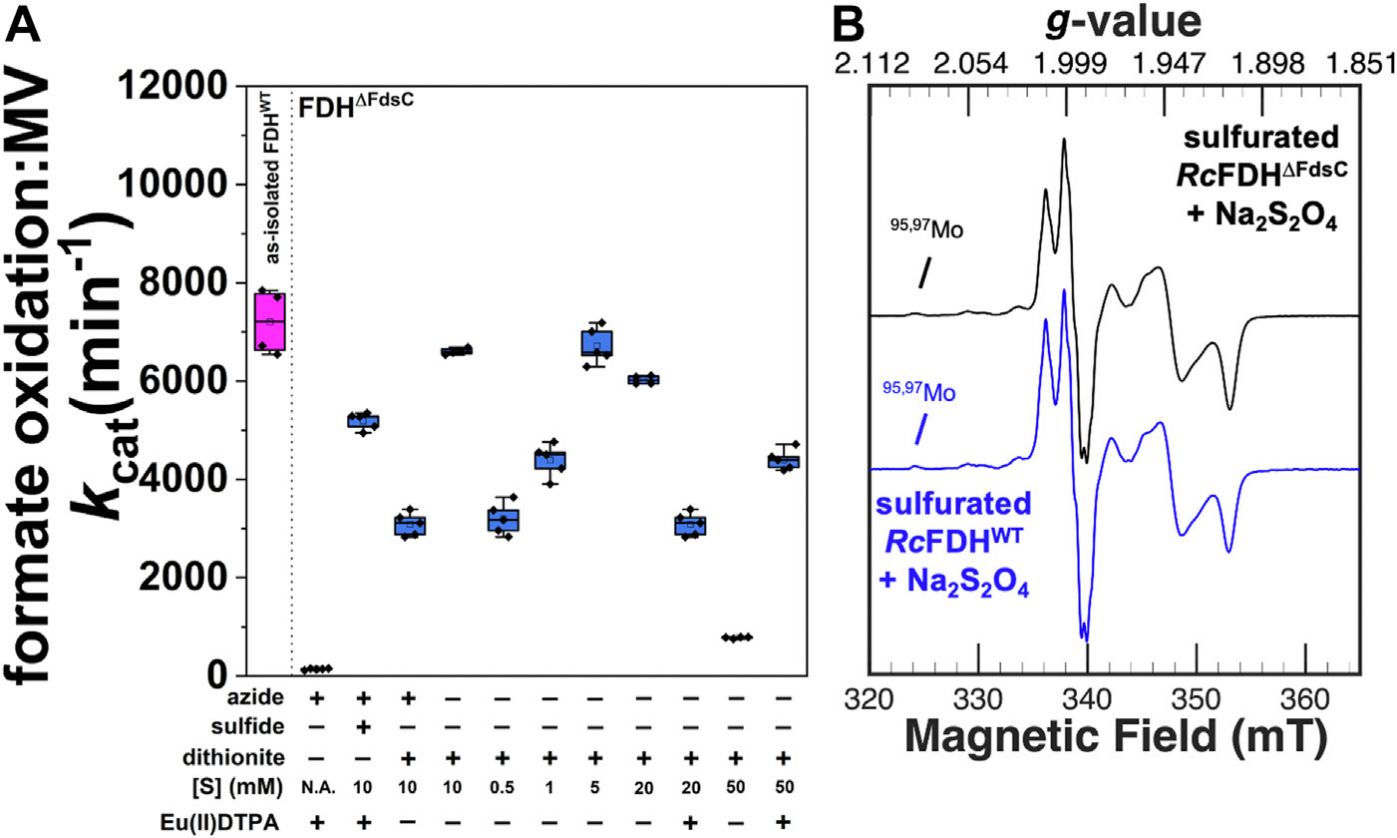
**The effect of sodium dithionite (Na_2_S_2_O_4_) on the *in vitro* sulfuration of *Rc*FDH^ΔFdsC^, as reflected in the formate oxidation *k*_cat_ catalyzed with oxidized methyl viologen as an electron acceptor.***A*, depicts the formate oxidation *k*_cat_ catalyzed with oxidized methyl viologen as an electron acceptor. The *blue* bars represent *in vitro* sulfurations of *Rc*FDH^ΔFdsC^, while the *magenta* bar represents as-isolated *Rc*FDH^WT^ as a control. Experiments reflect a 2.5 h incubation at 10 °C with the concentrations of above components (azide, dithionite or sulfide, and Eu^II^DTPA) listed, following workup and oxidative FMN treatment in 75 mM potassium phosphate, pH 7.5. Where applicable, the azide concentration during the sulfuration step was 10 mM. Formate oxidation activities are calculated with respect to the [Mo] present in the assay, and kinetics were measured with 400 *μ*M methyl viologen, 6 mM formate, and approximately 10 nM FDH in 100 mM tris–HCl, pH 9.0 buffer at ambient temperature. *B*, depicts EPR spectral characterization of the *Rc*FDH^ΔFdsC^ sample in (*A*) that was incubated with 20 mM Na_2_S_2_O_4_. The *black* and *blue traces* represent the sulfurated *Rc*FDH^ΔFdsC^ sample and the sulfurated *Rc*FDH^WT^ reference reduced with 10 mM Na_2_S_2_O_4_, respectively. Spectra were obtained at 110 K at 9.42 GHz microwave frequency, 4 mW microwave power, 2 G modulation amplitude, and 100 kHz modulation frequency. Each sample represents an [FDH] of 300 *μ*M. N.A. = not added.

Previous *in vitro* reconstitution of the active site sulfido ligand of XO was shown to be successful in the presence of only dithionite without the addition of another sulfur source (9). Dithionite can simultaneously act as a source of sulfur oxyanions and sulfide and as a reducing agent under anaerobic conditions (34, 35), while reducing *Rc*FDH^ΔFdsC^ with dithionite resulted in a distinct Mo^V^ species (Fig. S4). Sulfuration was, hence, performed in a fashion as described above, replacing both 10 mM Eu^II^DTPA and 10 mM Na_2_S by 10 mM Na_2_S_2_O_4_, in 75 mM potassium phosphate buffer, pH 7.5 with 10 mM NaN_3_ (Fig. 4). Similar to the sulfuration experiments with Eu^II^DTPA and sulfide, formate oxidation activity was observed that was roughly comparable to performing sulfuration with Eu^II^DTPA and Na_2_S in the presence of azide. Interestingly, efficient sulfuration also occurred with dithionite in the absence of azide (Fig. 4*A*). Based on these positive initial results, the efficiency of sulfuration with Na_2_S_2_O_4_ in the absence of azide was investigated in greater detail. Sulfurated *Rc*FDH^ΔFdsC^ similarly active as *Rc*FDH^WT^ was observed with a dithionite concentration range of 5 mM to 20 mM (Fig. 4*A*). When quantifying the relative activity of FMN reduction by formate substrate, the respective results were well comparable between *Rc*FDH^WT^, *Rc*FDH^ΔFdsC^ sulfurated with sulfide and Eu^II^DTPA, and respective sulfuration with dithionite (Fig. S7). Furthermore, when setting the formate oxidation activity into the context of the bis-MGD content detected as Form A-GMP, dithionite-sulfurated *Rc*FDH^ΔFdsC^ samples appeared even more active than *Rc*FDH^WT^, since a decreased Form A-GMP content in the former relative to the latter is observed, due to the absence of the chaperone FdsC (Fig. S8). To confirm the optimized apparent sulfuration of *Rc*FDH^ΔFdsC^ with dithionite, a sample was prepared specifically for EPR spectroscopy with the sequence (i) sulfuration with 20 mM Na_2_S_2_O_4_, (ii) work-up, and (iii) further or re-reduction with 10 mM Na_2_S_2_O_4_. An EPR spectrum of such sample was recorded at 110 K in the presence of 10 mM NaN_3_ (Fig. 4*B*). The detected Mo^V^ signal was identical to that of *Rc*FDH^WT^, with a negligible overlapping signal arising from the dithionite-reduced oxo bis-MGD cofactor (Fig. 4, see also Fig. 2*C*). By comparison, simple aerobic treatment of *Rc*FDH^ΔFdsC^ with 10 mM Na_2_S_2_O_4_ for 30 min at ambient temperature in the presence of 10 mM NaN_3_ resulted also in the dithionite-reduced Mo^V^ species associated with an oxo species, confirming that sulfuration with dithionite as a sulfur source is O_2_-sensitive (Fig. S9).

**Figure 5:**
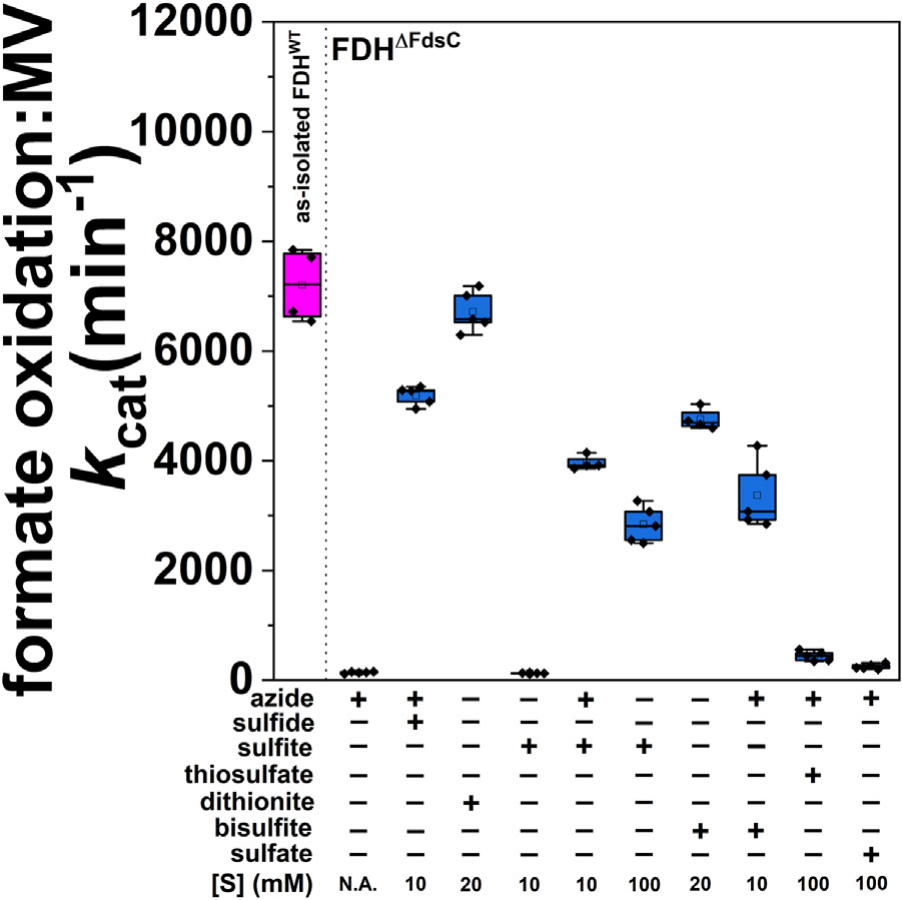
**The effect of associated byproducts of Na_2_S_2_O_4_ on the *in vitro* sulfuration of *Rc*FDH^ΔFdsC^.** The *blue bars* represent *in vitro* sulfurations of *Rc*FDH^ΔFdsC^, while the *magenta* bar represents as-isolated *Rc*FDH^WT^ as a control. Experiments reflect a 2.5 h incubation at 10 °C with the concentrations of above components (azide, dithionite, or sulfur-based decomposition products of dithionite) listed, following workup and oxidative FMN treatment. Where applicable, the azide concentration during the sulfuration step was 10 mM, and for all samples by which dithionite was not added, Eu^II^DTPA was added to a concentration of 10 mM. Formate oxidation activities are calculated with respect to the [Mo] present in the assay, and kinetics were measured with 400 *μ*M methyl viologen, 6 mM formate, and approximately 10 nM FDH in 100 mM tris–HCl, pH 9.0 buffer at ambient temperature. N.A. = not added.

Because incubation of *Rc*FDH^ΔFdsC^ with sodium dithionite under anaerobic conditions resulted in the insertion of a sulfido ligand, an active sulfur component in dithionite was likely responsible for the efficient sulfuration observed. Therefore, *in vitro* incorporation of a sulfido ligand at the active site of *Rc*FDH^ΔFdsC^ was attempted with alternative sulfur sources as possible byproducts of dithionite hydrolysis, employing Eu^II^DTPA as a sulfur-free reductant (Fig. 5). Interestingly, (bi)sulfite was a competent sulfur source, resulting in a comparable formate oxidation activity per MGD relative to using 10 mM sulfide as a sulfur source and was similar to sulfuration with dithionite (Fig. 4*A*). To confirm that dithionite and bisulfite are very closely related with regard to this specific chemical reactivity, *Rc*FDH^ΔFdsC^ was reduced with Eu^II^DTPA in the presence of 100 mM NaHSO_3_, and the Mo^V^ signal was assessed by EPR spectroscopy. Interestingly, the addition of bisulfite resulted in a nearly identical Mo^V^ signal to that from the reduction of *Rc*FDH^ΔFdsC^ with 10 mM Na_2_S_2_O_4_ (Fig. S10). By comparison, applying sulfate and thiosulfate as a sulfur source resulted in a partially more active enzyme compared to when no sulfur source was added. However, sulfite is a known starting material for the formation of thiosulfate (36). Possibly unreacted sulfite reagent as a contaminant contributes to the low apparent activity detected; however, these highly oxidized anions were in principle unable to act as a sulfur source (Fig. 5).

### Time-dependent sulfuration/resulfuration of *Rc*FDH^ΔFdsC^ and *Rc*FDH^WT^

**Figure 6:**
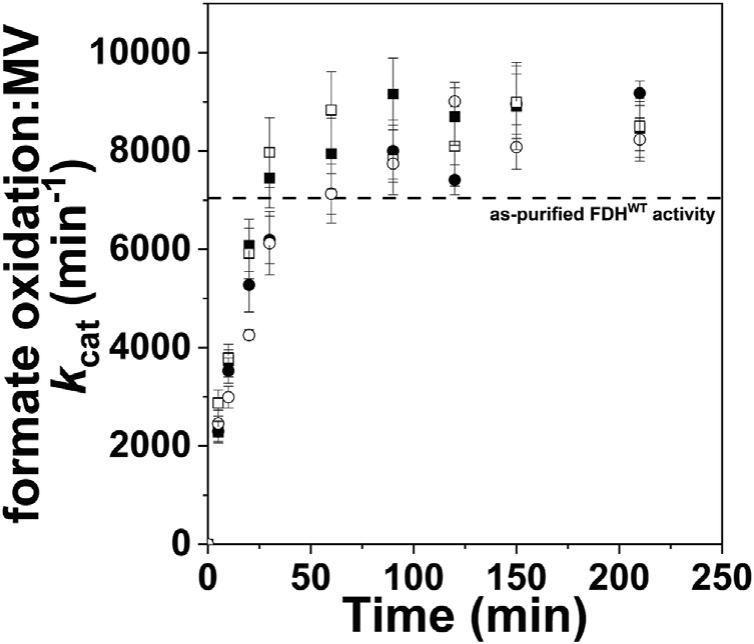
**Time-dependent formate:methyl viologen activity accompanying *in vitro* sulfuration of *Rc*FDH^ΔFdsC^ and cyanide-treated *Rc*FDH^WT^.** Either 35 *μ*M *Rc*FDH^ΔFdsC^ or 40 *μ*M cyanide-treated *Rc*FDH^WT^ was incubated at 10 °C in the absence of azide in the presence of either 10 mM Na_2_S_2_O_4_ or 20 mM NaHSO_3_ with 10 mM Eu^II^DTPA, with timepoints representing sampled aliquots from the reaction mixture. Squares represent *Rc*FDH^ΔFdsC^ and circles represent cyanide-treated *Rc*FDH^WT^; filled symbols represent treatment with Na_2_S_2_O_4_, and open symbols represent treatment with NaHSO_3_ and Eu^II^DTPA. At each timepoint, enzyme stock underwent a brief dilution and concentration with 75 mM potassium phosphate, 10 mM NaN_3_, pH 7.5 to stop the reaction. Each timepoint represents measured activity with 6 mM formate, 100 *μ*M methyl viologen in 100 mM tris–HCl, pH 9.0 buffer measured at ambient temperature, with approximately 2 nM FDH present, measured at ambient temperature (21 °C).

The reductive *in vitro* sulfuration of *Rc*FDH^ΔFdsC^ with sulfide, sulfite, or dithionite can be expected to be time-dependent as was described previously for XO/XDH (4, 9). This putatively reflects the combination of events such as reducing the bis-MGD cofactor, converting (bi)sulfite and/or dithionite to sulfide, and/or coordinating sulfide and/or (bi)sulfite to the active site (Fig. 3*A*). To compare the time dependence of *Rc*FDH^ΔFdsC^ sulfuration and of *Rc*FDH^WT^ resulfuration at the bis-MGD cofactor, time-course measurements were performed to assess the rate of activation as a consequence of sulfuration (Fig. 6). As is reflected in each curve, a slow increase in apparent formate oxidation activity was observed for *Rc*FDH^ΔFdsC^ and cyanide-treated *Rc*FDH^WT^ upon incubation with either 10 mM Na_2_S_2_O_4_ or 20 mM NaHSO_3_ with 10 mM Eu^II^DTPA (Fig. 6). After 5 min of incubation, cyanide-treated *Rc*FDH^WT^ exhibited approximately 33% of the as-isolated, azide-purified activity prior to cyanolysis, while *Rc*FDH^ΔFdsC^ exhibited comparable formate oxidation *k*_cat_ values with methyl viologen as an electron acceptor. After about 100 min incubation time, the change in formate oxidation *k*_cat_ reached a plateau of approximately 9000 min^−1^. Relative to the as-isolated *Rc*FDH^WT^ enzyme, the slight improvement in activity was consistent with the expected relative sulfuration state of the bis-MGD cofactor, following aerobic purification in the presence of azide (15). This shows that the cyanolyzed enzyme only lost the sulfido ligand, so that the enzyme could be almost fully reactivated by the chemical sulfuration procedure.

The ability of either Na_2_S_2_O_4_ or bisulfite to serve as a sulfur source of FDH is complicated by the requirement of a strong reductant, which could reflect an additional, exogenous reduction to generate sulfide. To assess this possibility, mixtures either in the presence or absence of FDH were assayed for sulfide over the course of sulfuration with the fluorescent, sulfide-binding compound 4-chloro-7-nitrobenzofurazan (NBD-Cl) (37). Either 10 mM Na_2_S_2_O_4_ or 10 mM Eu^II^DTPA with 10 mM NaHSO_3_ added to 80 *μ*M NBD-Cl resulted primarily in generation of the thioether NBD_2_S at 413 nm, with formation of the NBD-SH thiol at 534 nm as a minor species, representing approximately 20 *μ*M total sulfide detected in the absence of FDH that was not substantially different in the presence of FDH (Fig. S11). This amount did not change with respect to incubation time. In the absence of reductant and FDH, approximately 1 *μ*M sulfide was present in the bisulfite stock (data not shown). By comparison, in the presence of *Rc*FDH^ΔFdsC^ and over the course of sulfuration, a similar amount of approximately 20 *μ*M sulfide was detected that did not change with incubation time, despite obtaining catalytically competent enzyme for formate oxidation (Fig. S11). These observations suggest that the sulfuration actions of sulfide and dithionite and/or bisulfite likely constitute a separate, but cumulative effect of both.

### Characterization of ^33^S-sulfurated *Rc*FDH bis-MGD cofactor

**Figure 7:**
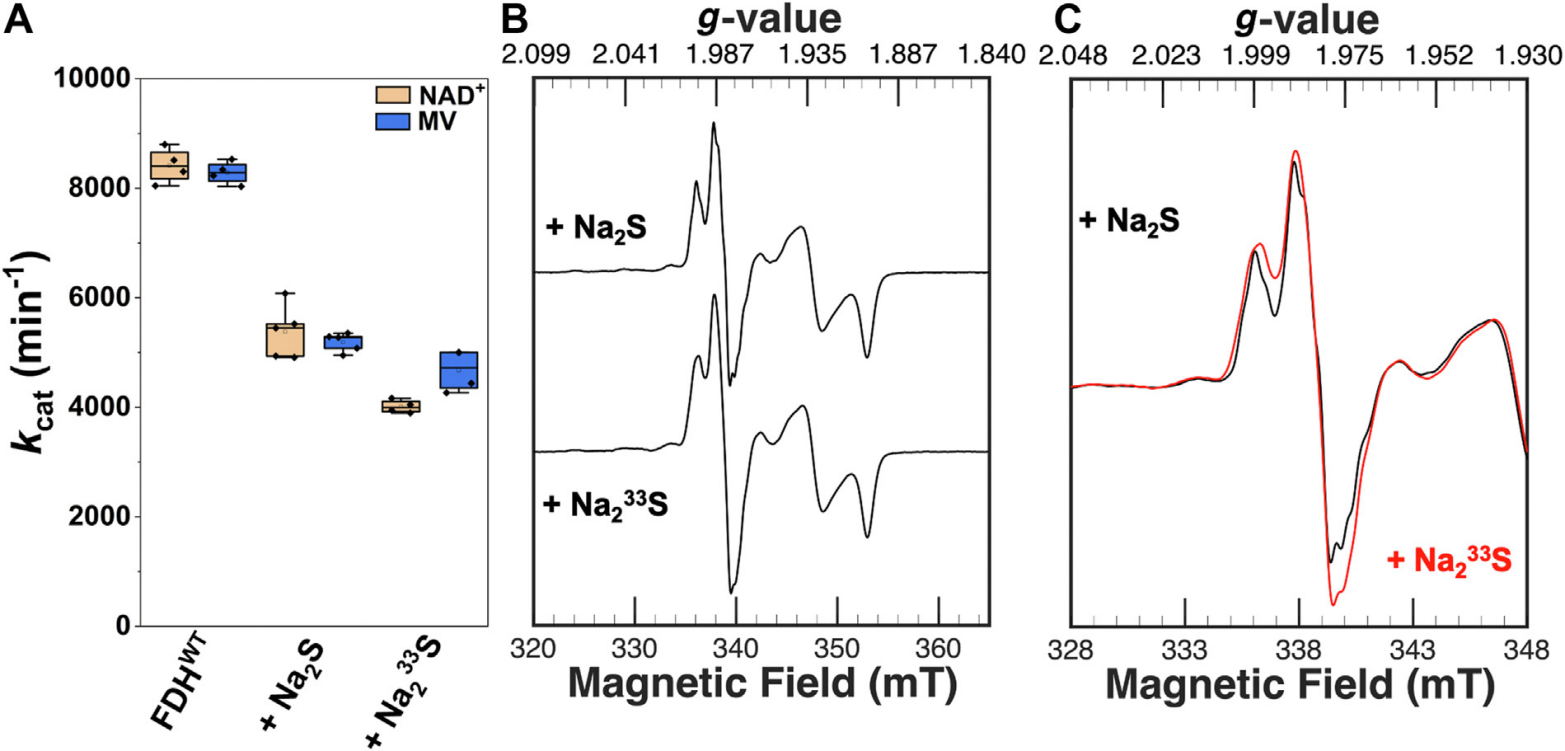
**Comparison of *in vitro* sulfuration of *Rc*FDH^ΔFdsC^ with Na_2_^33^S as a sulfur source.***A*, depicts formate oxidation activity measurements comparing sulfurated *Rc*FDH^WT^ with *in vitro* reconstituted *Rc*FDH^ΔFdsC^ with either natural abundance Na_2_S or Na_2_^33^S as a sulfur source with the electron acceptors NAD^+^ and oxidized methyl viologen. *B* and *C*, depict CW X-band EPR spectra of reconstituted *Rc*FDH^ΔFdsC^ samples with natural abundance Na_2_S or Na_2_^33^S reduced with 10 mM formate, with (*C*) depicting a zoomed-in overlay view of the ^1^H/^33^S hyperfine feature on the Mo^V^ species. *In vitro* sulfuration was performed in 75 mM potassium phosphate, 10 mM NaN_3_, pH 7.5 with Na_2_S and 10 mM Eu^II^DTPA, followed by oxidative treatment with K_3_[Fe(CN)_6_] and FMN reconstitution.

To assess the electronic role of the sulfido ligand at the active site of *Rc*FDH, *in vitro* sulfuration of *Rc*FDH^ΔFdsC^ with Na_2_^33^S was performed at pH 7.5, in the presence of Eu^II^DTPA and 10 mM NaN_3_ as described above. As expected, formate oxidation activity was observed with either NAD^+^ or oxidized methyl viologen as electron acceptors, which was comparable to the activity of natural abundance sulfurated *Rc*FDH^ΔFdsC^ (Fig. 7*A*). The formate-reduced spectrum in the presence of azide is shown in Figure 7*B*. The Mo^V^ signal was found to be highly similar to that of the natural abundance sulfurated enzyme, but with a slight broadening, reflecting unresolved hyperfine interactions. Spectral simulation of the CW X-band EPR spectrum showed an adequate reproducibility of the spectrum by including a ^33^S nucleus (Fig. 7*C*, see also Fig. S12). A first estimation of the hyperfine coupling yields 11.1, 4.2, and 10.2 MHz (Table 1, see also Table S1). Relative to the natural abundance sulfido ligand, an altered H-strain broadening and greater uncertainty in ^1^H hyperfine parameters were observed with the ^33^S sulfido ligand. Nevertheless, the magnitude of the ^33^S hyperfine feature was reminiscent to the “rapid type 1” and “rapid type 2” Mo^V^ species of XO and distinct from the “very rapid” signal, thus suggesting a Mo^V^–SH type interaction (38, 39, 40) and thereby confirming the presence of the chemically inserted sulfido ligand.

**Table 1:** Comparison of ^33^S hyperfine parameters in FDH and in other molybdoenzymes

Enzyme	Mo^V^ form	*A*_1,2,3_ (MHz)	*A*_av_ (MHz)	Reference
*Rhodobacter capsulatus* FDH	Sulfurated, azide-inhibited	11.2, 4.2, 10.2	8.5	This work
Bovine XO	Very rapid, xanthine	8, 78, 20	35	(39, 40)
	Very rapid HMP	9, 82, 16	36	(39, 40)
	Rapid type 1 formamide	10, 10, 10	10	(40)
	Rapid type 2 borate	10, 6, 10	9	(39)
Human SO	Low-pH	3, 2, 4	3	(53)
Human SO (R160Q)	Blocked (sulfite)	3.7, 4.6, −2.0	2.1	(54)

## Discussion

Several molybdoenzymes and tungstoenzymes including but not limited to FDH depend on a coordinative sulfido ligand for respective catalysis. Here, we have shown for the complex, iron–sulfur flavoenzyme FDH from *R. capsulatus*, that specific and unitary *in vitro* sulfuration of the bis-MGD cofactor can be achieved in the presence of a sulfur source and a reducing agent. By comparison to sulfuration of the well-studied enzyme XO/XDH (20), *in vitro* sulfuration of FDH meets and can exceed the sulfuration achieved from FDH biosynthesis. These procedures differ substantially from the prior work described for *Methanobacterium formicicum* FDH and for *E. coli* FDH (FdhF) (18, 24). In contrast to the former’s previous characterization, it can be confirmed that the selected conditions described (either with dithionite or with dithionite and sulfide) can sulfurate the FDH bis-MGD cofactor (18). However, given substantial differences in O_2_ sensitivity of FDHs and previous conductance of the sulfuration assay in the absence of azide, possibly cyanide treatment affected the ability of methyl viologen to bind to the enzyme or altered the Fe-S clusters bound (18). Cyanolysis of *Rc*FDH^WT^ resulted in quantified thiocyanate that was comparable, relative to the quantified molybdenum content and to the expected amount of sulfido ligand at the active site established previously by X-ray absorption spectroscopy (15), and was also similar to *M. formicicum* FDH (18). Regarding *in vitro* sulfuration of *E. coli* FdhF, a modest stimulated formate oxidation activity was previously observed upon incubation with sodium sulfide in the absence of reductant activity of the *E. coli* FdhF active site variant U140C (24). However, differences in the requirement of reducing agent to afford sulfuration can be potentially explained by differences in the oxidation state of the bis-MGD cofactor, in that it was likely already reduced to the Mo^IV^ oxidation state (24). Furthermore, it should be noted that while cyanolysis of the sulfide-activated FdhF^U140C^ active site variant did not result in a complete loss of activity, considering the p*K*_a_ of HCN and the buffer pH, likely less cyanide was present than was expected (24). Previously, we have shown that the Mo ion in *Rc*FDH^ΔFdsC^ in the as-isolated state and in *Rc*FDH^WT^ following treatment with cyanide in the as-obtained state is in the Mo^VI^ state, consistent with an oxidized cofactor (14, 15). Similarly, successful sulfuration requires a reducing agent in conjunction with a sulfur source, which is similar to well-established sulfuration methods described for XO.

Differences in the *g-*tensor and spectral shape in the oxo-containing Mo^V^ and conversion to a sulfido-containing Mo^V^ provide direct experimental evidence for sulfuration. This is additionally corroborated by the broadening of the Mo^V^ EPR spectrum when Na_2_^33^S is used for sulfuration. As was emphasized elsewhere (41), the electronic environment around the bis-MGD cofactor Mo^V^ ion is largely dependent on the coordination environment and the ligands that surround it. In this work, differences in behavior of the coordination environment are observed upon extended reductive treatment of the incompetent oxo-bis-MGD cofactor with a sulfur source, as reflected in the course of the sulfuration assay. Reduction and prompt freezing of *Rc*FDH^ΔFdsC^ with dithionite yields a distinct Mo^V^ species, which is presumed to reflect an oxo-bis-MGD cofactor with a deprotonated O^–^ ligand replacing Cys386. This signal is similar to the Mo^V^ species detected for *E. coli* DMSO reductase variant S176A (42), is reminiscent to a Mo^V^ state reported for FDHs from *Pseudomonas aeruginosa* (43) and later from *Desulfovibrio desulfuricans* (both FDHs possess an active site selenocysteine residue) (44, 45), and is also analogous to the rhombic Mo^V^ signal reported for the nitrate reductase NarGHI complex from *Marinobacter hydrocarbonoclasticus* strain 617 (46). In each case, the generated Mo^V^ signal appears to be independent of the active site residue bound at the active site and by extension is different from the azide-inhibited sulfurated Mo^V^ state. By comparison, sulfuration of *Rc*FDH^ΔFdsC^ results in an altered Mo^V^ species of increased *g*_av_ and decreased *g*_aniso_ exhibiting ^1^H-hyperfine coupling that is consistent with a MoS_6_ coordination geometry with the coordinated sulfido ligand and the active site Cys ligand bound to the molybdenum center (31, 32).

*In vitro* sulfuration of *Rc*FDH is an exquisitely O_2_-sensitive reaction requiring strictly anaerobic conditions. Substantial incorporation of the sulfido ligand with sulfide can be facilitated in the presence of the inhibitor azide, which itself protects the sulfido ligand from oxidative damage and inhibits FDH formate oxidation and CO_2_ reduction *via* binding in the second coordination sphere (15, 16). Herein, it was shown that as long as the sulfuration buffer is strictly anaerobic, comparable sulfide-based sulfuration in the absence of azide relative to *Rc*FDH^WT^ isolated in the presence of azide is achievable. These results are consistent with the successful *in vitro* sulfurations with dithionite or with bisulfite plus Eu^II^DTPA, which do not require azide co-incubation for efficient sulfido ligand incorporation. Dithionite and bisulfite are also known O_2_ scavengers to produce bisulfite and/or bisulfate, respectively, while removing O_2_ *in situ* (36, 47, 48). Previously, it was shown for *Cupriavidus necator* FDH that sulfite is produced upon superoxide oxidation of the active site sulfido ligand (49). Such oxidation actually constitutes the reverse reaction for the transformations explored herein. It can therefore be confirmed that sulfite may be used as a sulfur source for the sulfido ligand at the bis-MGD active site.

**Figure 8:**
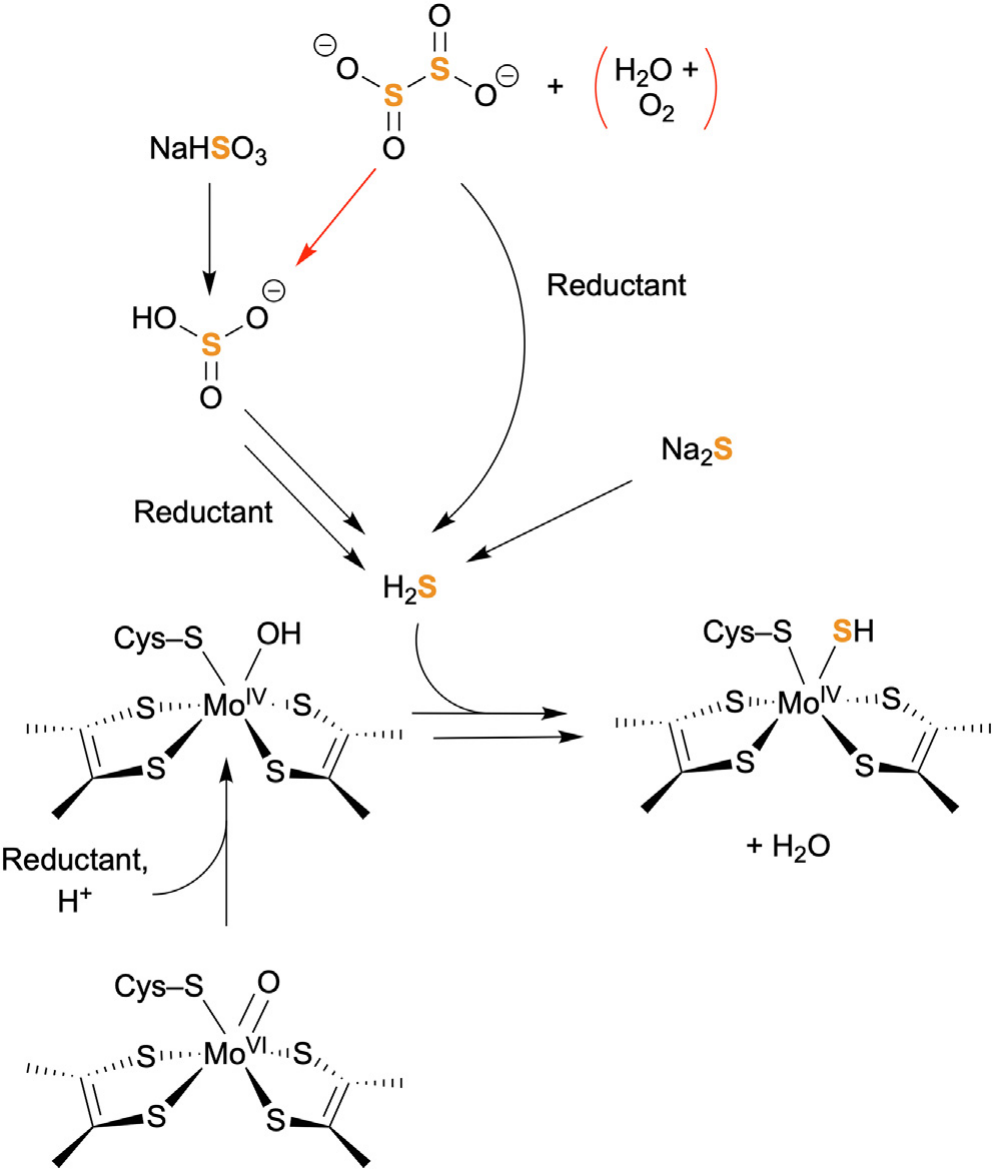
**Model for *in vitro* sulfuration of the bis-MGD cofactor by *Rhodobacter capsulatus* FDH, based upon the experimental data presented herein.** Installation of a sulfido ligand on the catalytically incompetent oxo-bis-MGD cofactor present in *Rc*FDH^ΔFdsC^ requires an electron source (such as Eu^II^DTPA or Na_2_S_2_O_4_), a sulfur source (such as sodium sulfide, sodium bisulfite, or Na_2_S_2_O_4_), and buffer conditions below pH 8.0. The reducing agent is likely required to alter the bond order of the bound oxo ligand to form a hydroxo ligand in the Mo^IV^ oxidation state, resulting in exchange with excess hydrosulfide or H_2_S present. The efficiency of sulfuration, particularly with sulfide as a sulfur source, is O_2_-sensitive, which can be mitigated either by strict anaerobic technique or by the addition of the inhibitor azide, that protects the sulfido ligand *in situ*. Na_2_S_2_O_4_ is a known O_2_ scavenger that can react to form bisulfite as a potential byproduct, removing the unwanted O_2_ present (as depicted in *red arrows*). Dithionite also can produce sulfide directly. Given the observation that strong reductants such as Na_2_S_2_O_4_ or Eu^II^DTPA are present, bisulfite serves as an alternative sulfur source and can undergo reduction to form sulfide. Installation and oxidation of the bis-MGD cofactor results in a doubly-bonded sulfido ligand at the active site.

*Rc*FDH’s promiscuous ability to incorporate a sulfido ligand *in vitro* with either sulfide or (bi)sulfite as a sulfur source, along with XO/XDH, potentially suggests multiple routes of sulfuration of the active site, considering the distinct oxidation states of the sulfur sources (Fig. 8). On one hand, direct reductive sulfuration with the reduced sulfur source sulfide, that is inactive in the absence of a reductant, shows that only a reduced bis-MGD cofactor is able to receive the chemically supplied sulfide to bind it as sulfido ligand. By comparison, (bi)sulfite and/or dithionite are apparently reduced with the low potential electrons provided by dithionite or Eu^II^DTPA (50) to generate sulfide for its installation at the bis-MGD cofactor. This mechanism could account for a common sulfuration approach across the identified sulfur sources, that is supported by the low, but constant sulfide concentration detected. On the other hand, direct coordination and/or binding of (bi)sulfite to the oxo bis-MGD, followed by a series of two-electron reduction events, would provide a way to generate sulfide *in situ* at the active site. This would be a mechanism reminiscent of dissimilatory sulfite reductases (51) or nitrogenases (52), that is also supported by the low, but constant sulfide concentration detected. It may be possible that oxyanions such as (bi)sulfite do bind at the active site of FDH, since other molybdoenzymes are known to catalyze oxygen atom transfer reactions involving such substrates (2). These details as relevant to FDH await future study.

The highly identical Mo^V^ *g-*tensor and hyperfine parameters between FDH undergoing sulfuration in the presence of FdsC/FdhD and *in vitro* sulfuration depicted herein support a comparable MoS_6_ coordination environment for the Mo^V^ oxidation state with the sulfido ligand binding a solvent-exchangeable proton as observed elsewhere (15, 32). The magnitude of the ^33^S hyperfine coupling in *Rc*FDH^ΔFdsC^ sulfurated with Na_2_^33^S exhibits a similar strength as the “rapid” and inhibited Mo^V^ species of XO (38, 39, 40) and as the *hfi* of ^33^S-labeled MPT detected as part of the low-pH Mo^V^ species in bioengineered human SO (53) as well as of the ^33^S substrate-blocked form of bioengineered human SO (54). All of those were shown to be associated with an elongated Mo–S-type bond.

The apparent modification of the bis-MGD cofactor in FDH with dithionite to yield a bound sulfido ligand offers relevance to the active site characterization of bis-MGD coordinating molybdoenzymes, with respect to the underappreciated role played by the sulfido ligand that has largely escaped detection as being mechanistically relevant. Several bis-MGD enzymes catalyzing oxygen atom transfer reactions depend on co-incubation with dithionite, either to redox-cycle or reductively activate the enzyme (55, 56), whereas at the same time, involvement of a sulfido ligand was excluded in the past. In contrast, other bis-MGD–containing enzymes have been identified to have a sulfido ligand at their active site, including the periplasmic nitrate reductase from *C. necator* (57), the homologous nitrate reductase from *D. desulfuricans* ATCC 27774 (58), and TMAO reductase from *E. coli* (59), although the mechanisms for sulfuration of their respective cofactors await further characterization. There are notable similarities with regard to the nitrogenases, for which the ninth sulfur in the complex iron-sulfur cluster active site was shown to stem from sulfite, which was present in the reductant dithionite employed as part of purification methods (52). More recently, similar observations were made for the bis-W-MPT enzyme aldehyde ferredoxin oxidoreductase (60, 61), which also is commonly purified anaerobically in the presence of dithionite as a reductant (62, 63). Its coordination sphere, very recently, was scrutinized for the likely inclusion of a sulfido ligand (64). Herein, the incubation of incompetent *Rc*FDH^ΔFdsC^ with dithionite for 30 min at 4 °C under anaerobic conditions is sufficient to activate the enzyme for formate oxidation, reflecting dithionite’s ability to sulfurate the bis-MGD cofactor. While the sulfido ligand is accepted as a critically important coordinating ligand to the bis-MGD Mo/W ion in FDH, resistance against acceptance of a nascent sulfido ligand as mechanistically relevant persists for other bis-MGD–containing enzymes. This is the case, for example, for nitrate reductase from *Campylobacter jejuni* (65) and despite evidence for the sulfido ligand as relevant for optimal catalysis for the TMAO reductase from *E. coli* (59). Revisiting coordinative binding of the sulfido ligand at other bis-MGD enzymes to confirm the mechanism of action awaits future effort.

## Conclusion

The results of this work demonstrate the interplay and the common mechanisms for active site sulfuration at the active sites in the XO/XDH and DMSO reductase families of molybdoenzymes and tungstoenzymes. Analyzing the mode of *in vitro* sulfuration of a DMSO reductase family molybdoenzyme for which participation of the active site sulfido ligand is accepted, these experiments provide a basis for assessing other metalloenzymes catalyzing an OAT reaction for the presence of a sulfido ligand. This is true even for those without apparent association of maturation enzymes identified to biosynthetically install a sulfido ligand. This work thereby facilitates access to a potential platform for addressing aspects of the activity of molybdoenzymes and tungstoenzymes, the function or catalytic activity of which is not (fully) established as of yet. The demonstration that *in vitro* sulfuration can match or exceed that accomplished in course of biosynthetic enzyme maturation provides an important benchmark by which targeted *in vitro* sulfuration can be applied in other enzymatic systems featuring difficult overexpression hosts. Routinely, metalloenzymes are treated with sodium dithionite for spectroscopic characterization or protein purification, which results in an altered protein activity and active site environment. Sulfide, dithionite, and (bi)sulfite for either XO/XDH or FDH are not identified as substrates for catalysis. However, considering that molybdoenzymes and tungstoenzymes catalyze key reactions in sulfur, nitrogen, and carbon cycling and that some of the enzymes of this family catalytically consume or produce (bi)sulfite and sulfide, it is possible that these reagents can in fact react at the active site, such as with a catalytically incompetent oxo ligand in the presence of reductant and in the absence of O_2_.

## Experimental procedures

### Chemicals and equipment

The chemicals and reagents used in this study were generally obtained from commercial sources and were of the highest purity. Peptone, yeast extract, plant agar, IPTG, Tris, chloramphenicol, and sodium phosphate were obtained from Duchefa Biochemie. Protino Ni nitrilotriacetic acid agarose was obtained from Macherey-Nagel. Potassium phosphate and 2-(*N-*morpholino)ethanesulfonic acid was obtained from Merck Chemicals. Sodium chloride, hydrochloric acid, sodium hydroxide, toluene, and potassium hydroxide were obtained from VWR chemicals. Imidazole, ampicillin, sodium sulfate, DMSO, and sodium azide were obtained from Carl Roth. Sodium formate was obtained from Fluka Biochemika. Elemental ^33^S_8_ was obtained from Eurisotop. Ultrapure elemental sodium was obtained from Thermo Fisher Scientific (>99.95%, metal trace basis). Europium(II) chloride, DTPA, FMN, sodium molybdate, sodium sulfide, sodium hydrosulfite (sodium dithionite), sodium sulfite, sodium bisulfite, sodium thiosulfate, ß-NAD^+^, 1,1′-dimethyl-4,4′-bipyridinium dichloride (methyl viologen) were obtained from Sigma–Aldrich. Ammonia was obtained from Air Liquide Deutschland. NBD-Cl was obtained from Alfa Aesar. The sodium dithionite used was stored aerobically but represented a “fresh” stock solid that was used within 1 year of opening. To ensure the quality of the dithionite used, freshly suspended dithionite stocks were routinely evaluated in their ability to reduce FDH relative to reduction with dithionite upon initial opening of the container, whereby comparable reduction of the enzyme was observed. The degassing and preparation of anoxic buffer used for sulfuration was achieved through standard Schlenk line techniques (66), using 500 ml to 1 L Erlenmeyer flasks equipped with a ground glass joint that could be connected to a Schlenk adaptor (Lenz Laborglas). Buffers were made anaerobic with iterative vacuo-pumping and purging with in-house N_2_ gas supplied without additional cleaning. Successful sulfuration attempts in the absence of azide were possible, as long as the buffer used for sulfuration was freshly degassed and as long as the MBraun anaerobic chamber did not exhibit elevated O_2_ levels. Ampoules of europium(II) chloride solid were opened, handled, and stored in a homemade minidesiccator with activated Blaugel 3 (VEB Laborchemie Apolda) within an MBraun anaerobic chamber in the absence of H_2_ and O_2_ (<10 ppm) to maintain usability. For quality control, the concentration of the Eu^II^DTPA was occasionally estimated *via* UV-visible absorbance spectroscopy, according to its two extinction coefficients reported elsewhere (30).

### Chemical synthesis of Na_2_^33^S

The synthesis of Na_2_^33^S was adapted and modified from a previous report (67). The sodium ampoule (as delivered) was opened under an inert argon atmosphere and transferred into dried and oxygen free toluene. After refluxing (melting the sodium at the boiling temperature of toluene) and re-cooling under vigorous stirring, small solid sodium beads were obtained. These sodium beads (54 mg, 2.3499 mmol, 2 eq.) were transferred inside an MBraun glove box into a 100 ml, 2-neck round bottom Schlenk flask and stuck to the glass wall applying a gentle push with a glass rod. The ^33^S (38.7 mg, 1.183 mmol, 1 eq.) was placed carefully on the opposite side of a pre-dried magnetic stirring bar in the same Schlenk flask. The two necks of the Schlenk flask were closed with one stopcock and one additional gas bubbler also closed with a stopcock. The sealed airtight Schlenk flask was moved to a Schlenk-line equipped with a Y-connector to allow for proper evacuation and addition of (dry) gaseous NH_3_ but first set under an argon atmosphere. The flask was cooled to approximately −50 °C in a cool bath. Slowly and carefully, the NH_3_ flow was opened while the argon flow was closed-off. The NH_3_ inflow was adjusted (avoiding under-pressure) to merely a small over-pressure using the bubbler for flow control. The NH_3_ condensed inside the cold Schlenk flask to become the reaction solvent resulting in an immediate dark-blue coloration upon contact with the sodium beads. The positioning of the Schlenk flask is then carefully adjusted to allow contact of the solution with the ^33^S, resulting in a slight shift towards red color upon contact. Immediate strong stirring allows for an essentially completely homogenous reaction while the level of liquid NH_3_ should reach and be retained at approximately 50 ml throughout the process. Upon full conversion of the sodium, indicated by obtaining a yellowish-milky mixture, the NH_3_ stream was stopped and a gentle Ar-flow was allowed to remove the evaporating NH_3_ while carefully warming to room temperature. After complete removal of NH_3_, the resulting Na_2_^33^S powder was dried *in vacuo* and transferred anaerobically into glass ampoules, which were then sealed. Yield: ∼87% (81.2 mg, 1.03 mmol).

### Expression and purification conditions

*R. capsulatus* formate dehydrogenase (*Rc*FDH) was heterologously expressed in *E. coli* under aerobic conditions and was purified as described previously, with minor modifications (15, 68). Briefly, expression of the holoenzyme possessing (*Rc*FDH^WT^) or lacking (*Rc*FDH^ΔFdsC^) the sulfido ligand was achieved *via* co-expression of plasmids pTHfds05 and pTHfds07, and pTHfds04 and pTHfds12 transformed in the *E. coli* MC1061 DE3 and BW25113 *ΔfdhD* DE3 cell lines, respectively, as described elsewhere (68). Similarly, isolation of the apoenzyme lacking the bis-MGD cofactor was achieved *via* expression of plasmid pTHfds05 in *E. coli* RK5200 *ΔmoaA* cells in the absence of molybdate, as described elsewhere (68). Harvested cells were suspended in lysis buffer (50 mM phosphate, 300 mM NaCl, 10 mM imidazole, pH 8.0) in the presence of 10 mM NaN_3_. Subsequent purification steps, including Ni nitrilotriacetic acid-based affinity chromatography, removal of the imidazole *via* Sephadex G-25 M PD-10 desalting columns (Cytiva), and size exclusion chromatography using a HiLoad 16/600 Superdex 200 pg column (Cytiva), were performed aerobically in the presence of 10 mM NaN_3_ at 4 °C. Aliquots constituting the isolated heterodimeric fraction were confirmed by SDS-PAGE and were pooled and concentrated with ultracentrifugation devices (Vivaspin 20, 50 kDa MWCO; Sartorius AG) in 75 mM potassium phosphate, 10 mM NaN_3_, pH 7.5 buffer. Concentrated stock FDH solutions representing 30 to 60 mg of isolated FDH protomer were flash-frozen in liquid N_2_ and were stored in a −80 °C freezer.

### Cyanolysis of competently active *Rc*FDH^WT^

Inactivation of *Rc*FDH^WT^ *via* removal of the active site sulfido ligand was achieved in an MBraun anaerobic chamber in the absence of H_2_ and O_2_ (<50 ppm), residing in a cold room at 4 °C, following literature procedure for XO/XDH (4, 9, 26, 28) and *M. formicicum* FDH (18) adapted with a few experimental changes. Starting from aerobically purified *Rc*FDH^WT^ in the presence of azide, 40 to 50 mg of *Rc*FDH^WT^ was exchanged into azide-free buffer (75 mM potassium phosphate, pH 7.5) *via* Sephadex G-25 PD-10 desalting columns, yielding approximately 5 ml of 30 *μ*M *Rc*FDH^WT^. An aliquot of this stock was reserved for formate oxidation kinetic assays (see Experimental procedures section “Activity measurements”). To 4 ml of desalted *Rc*FDH^WT^ was added 40 μl of freshly prepared 100 mM KCN (1 mM final concentration) in the above buffer, and upon mixing, incubated for 30 min at 10 °C. Aliquots were taken to assess time-dependent cyanide inactivation of FDH formate oxidation activity, but all samples were inactive upon dilution into 75 mM potassium phosphate, 10 mM NaN_3_, pH 7.5 buffer, similar to previous similar experiments (15). The cyanide-treated sample underwent concentration with Amicon Ultra-0.5 centrifugal filters (100 kDa MWCO) (Merck–Millipore). The flow-through was kept, and the concentrated, cyanide-treated enzyme underwent iterative PD-10 desalting into 75 mM potassium phosphate, pH 7.5 buffer. The enzyme stock at this stage was employed for resulfuration *via* the methods described below for *Rc*FDH^ΔFdsC^. The cyanide-derived thiocyanate from the reaction with the active site sulfido ligand was quantified *via* the Sörbo assay (69), as described previously (70), with some slight modifications. To 500 μl of the above flow-through (in 75 mM potassium phosphate, pH 7.5 buffer) was added 500 μl of Sörbo's reagent (165 mM Fe^III^ nitrate in 8.7% HNO_3_) and the mix incubated aerobically for 10 min at ambient temperature before the absorbance was recorded at 460 nm on a Shimadzu UV-160 A spectrophotometer. Analytical triplicate flow-through samples were assessed. A standardized curve of thiocyanate prepared in the above 75 mM potassium phosphate, pH 7.5 buffer was compared in parallel to the flow-through samples.

### *In vitro* sulfuration in the absence of dithionite

Sulfuration of *Rc*FDH^ΔFdsC^ or cyanolyzed *Rc*FDH^WT^ was performed in an MBraun anaerobic chamber in the absence of H_2_ and O_2_ (<50 ppm), residing in a cold room at 4 °C. Briefly, aliquots of aerobically purified, nonactive *Rc*FDH^ΔFdsC^ or cyanolyzed *Rc*FDH^WT^ (equating to approximately 30–60 mg FDH protomer) were equilibrated with degassed 75 mM potassium phosphate, 10 mM NaN_3_, pH 7.5 *via* iterative PD-10 desalting, yielding approximately 6 ml of diluted FDH stock. From this stock, to an aliquot of ∼1350 to 1500 *μ*l FDH was added 170 *μ*l of 120 mM Na_2_S and 170 *μ*l of 120 mM Eu^II^DTPA (prepared from a working stock of degassed 75 mM potassium phosphate, 200 mM DTPA, pH 7.5 stored in the MBraun chamber and dilution with DPTA-free buffer). Addition of Eu^II^DTPA resulted in an observable color change of the FDH sample from brownish-yellow to a faint yellow. The aliquot was then incubated at 4 °C for 2.5 h, whereby samples often were faintly cloudy with precipitation. Samples were then briefly centrifuged with an Eppendorf MiniSpin centrifuge housed in the MBraun chamber, prior to concentration with Amicon Ultra-0.5 centrifugal filters (100 kDa MWCO) (Merck–Millipore). To remove the excess Eu^II^DTPA and sulfide, concentrated samples underwent iterative PD-10 desalting into 75 mM potassium phosphate, 10 mM NaN_3_, pH 7.5 buffer. At this point, the reconstituted enzyme exhibits formate oxidation activity with oxidized 1,1′-dimethyl-4,4′-bipyridinium dichloride (methyl viologen) but does not show formate oxidation with NAD^+^ as an electron acceptor. To reconstitute enzymatic activity using NAD^+^ as an electron acceptor, to 1500 *μ*l of the desalted FDH was added 170 *μ*l of 12 mM ferricyanide and 170 *μ*l of dissolved FMN (10 mg/ml). Samples were incubated for 30 min at 4 °C prior to centrifugation and similar to reductive treatment above, were concentrated, underwent iterative PD-10 desalting, and were subsequently concentrated to a final working volume stock of 100 to 300 *μ*l in 75 mM potassium phosphate, 10 mM NaN_3_, pH 7.5. To assess the effect of sulfuration, a UV-visible absorbance spectrum was obtained immediately following sulfuration, which was performed aerobically on a benchtop Shimadzu UV-2600i UV-visible spectrophotometer (Shimadzu Europa) in the above 75 mM potassium phosphate, 10 mM NaN_3_, pH 7.5 buffer, which are typical conditions for the aerobic isolation of *Rc*FDH^WT^ with optimal retention of the sulfido ligand, as reported previously (15). The “as-sulfurated” spectrum was compared relative to the “as-obtained” spectrum of *Rc*FDH^ΔFdsC^, as well as relative to the as-obtained spectrum of the sulfurated *Rc*FDH^WT^. To assess the loading of the sulfido ligand in sulfurated FDH samples, formate was added (10 mM final concentration) and the resultant spectrum was compared relative to the as-sulfurated spectrum. Typical % yield of FDH after sulfuration (following oxidative FMN reconstitution using the methods employed above) was approximately 26%.

To assess the potential pH dependence on the sulfuration of *Rc*FDH^ΔFdsC^, the experiments were performed across a wide pH range, using either 100 mM 2-(*N-*morpholino)ethanesulfonic acid pH 5.5, 75 mM potassium phosphate pH 6.0 to 7.5, or 75 mM tris–HCl, pH 8.0 to 9.0 (each in the presence of 10 mM NaN_3_). The sulfuration step was performed in this buffer under the conditions described above, and subsequent handling steps, including desalting and oxidative FMN treatment, were performed in the usual 75 mM potassium phosphate, 10 mM NaN_3_, pH 7.5 buffer. Once optimal sulfuration conditions could be ascertained at different pH values, to compare the contribution of azide to the sulfuration, the sulfuration was repeated in selected buffers described above in the absence of azide, with subsequent steps performed in 75 mM potassium phosphate, 10 mM NaN_3_, pH 7.5 buffer, as in the above procedures.

### *In vitro* sulfuration in the presence of dithionite

Na_2_S_2_O_4_ is known both as a reducing agent and a sulfur source and was treated as such in the course of sulfuration. Methods for sulfurating *Rc*FDH^ΔFdsC^ or cyanide-treated *Rc*FDH^WT^ in the presence of Na_2_S_2_O_4_ generally were similar to the methods described above for dithionite-free sulfuration, with the simple replacement of Eu^II^DTPA and of Na_2_S with Na_2_S_2_O_4_ in 75 mM potassium phosphate, 10 mM NaN_3_, pH 7.5 buffer. However, more efficient sulfuration (yielding more active sulfurated enzyme) could be realized for FDH samples transferred into anaerobic azide-free buffer (75 mM potassium phosphate, pH 7.5) prior to sulfuration. After making the FDH sample anaerobic in the azide-free buffer, dithionite was added at different concentrations (500 *μ*M–50 mM Na_2_S_2_O_4_) and from this point onwards, was handled identically to the above azide-containing samples. Upon concentration of the reduced enzyme, PD-10 buffer exchange into the above azide-containing buffer (75 mM potassium phosphate, 10 mM NaN_3_, pH 7.5) was performed, and all subsequent steps were performed in an identical fashion to dithionite-free sulfuration assays.

Hybrid sulfuration assays to identify the sulfur source in sodium dithionite were performed in the presence of the reductant Eu^II^DTPA along with identified components present in dithionite upon dissolving in aqueous buffer (34, 35, 36) using the methods described above for dithionite-free sulfuration and with different sulfur source concentrations. Specific concentrations of sulfur source, reducing agent, and the absence or presence of azide are provided in the respective figure legends.

### Sulfide detection

Assays to detect apparent sulfide in *in vitro* sulfuration reactions in the presence of Na_2_S_2_O_4_ or bisulfite in the presence of Eu^II^DTPA were adapted from the assays described elsewhere (37). Reactions of 550 μl volume were prepared in an MBraun anaerobic chamber (<10 ppm O_2_) containing 75 mM potassium phosphate, pH 7.5 that also contained 10 mM Na_2_S_2_O_4_ or 10 mM NaHSO_3_ and 10 mM Eu^II^DTPA, in the absence and presence of 32 *μ*M *Rc*FDH^ΔFdsC^, 24 *μ*M *Rc*FDH^WT^, or 22 *μ*M *Rc*FDH^Δbis-MGD^. Samples incubated at 10 °C, and the addition of reductant started the reaction. To one set of samples after about a minute of incubation, the FDH samples were centrifuged, and to 500 *μ*l of flow-through, 4 *μ*l of 10 mM NBD-Cl (dissolved in DMSO) was added, and samples were made aerobic. The retained enzyme was saved for kinetic measurements. After about 30 min of incubation, samples were diluted 1:6.7 with distilled water and the absorbance at 342, 413, and 534 nm was measured for each sample. The same procedure was followed for another set of samples after 60 min and 150 min incubation at 10 °C. All samples were faint yellow in color. UV-vis measurements were performed in triplicate on a Shimadzu UV-160A spectrophotometer. The sulfide concentration was estimated from the cumulative absorbance of the NBD_2_S thioether at 413 nm (ε = 11,700 ± 100 M^−1^ cm^−1^) and the NBD–SH thiol at 534 nm (ε = 19,000 ± 600 M^−1^ cm^−1^) (37). For calibration, a standard curve was prepared of 1 to 250 *μ*M Na_2_S dissolved in 75 mM potassium phosphate, pH 7.5, whereas NBD-Cl was added and the absorbance at the stated wavelengths were recorded. The isolated FDH was assessed for formate oxidation *via* the methods described below. Sulfide detection was also assessed for 10 mM NaHSO_3_ in the above buffer in the absence of reductant and was found to be approximately 1 *μ*M.

### Activity measurements

*Rc*FDH formate oxidation activity (requiring the active site sulfido ligand) was measured following the rate of generating reduced 1,1′-dimethyl-4,4′-bipyridinium dichloride (methyl viologen) (ε_578 nm_ = 9.70 mM^−1^ cm^−1^) or NADH (ε_340 nm_ = 6.22 mM^−1^ cm^−1^) with a Shimadzu 1280 spectrophotometer (Shimadzu Europa) housed in an anaerobic Coy chamber (Coy Laboratory Products) (O_2_ < 10 ppm) similar to previously published kinetic measurements, but with slight differences (16, 71). Briefly, more diluted FDH stock solutions were employed for measurements, with an FDH end concentration of 5 to 10 nM. Diluted stock solutions were prepared from concentrated stocks in 75 mM potassium phosphate, 10 mM NaN_3_, pH 7.5 buffer. In activity assays, the residual azide concentration was approximately 50 *μ*M, which is approximately equal to the azide *K*_i_ value inhibiting formate oxidation. Activity measurements were typically performed five to six times per measurement with 6 mM sodium formate and either 400 *μ*M methyl viologen or 2 mM NAD ^+^ as the electron acceptor in 100 mM Tris–HCl, pH 9.0 buffer. Following activity measurements, the concentration of FDH, [FDH], was routinely confirmed with a Shimadzu BioSpec-nano spectrophotometer (Shimadzu Europa). Where applicable, one unit of FDH activity (U) is defined as 1 *μ*mol of formate oxidized per minute. Because expression of *Rc*FDH^ΔFdsC^ was performed in the *E. coli* BW25113 *ΔfdhD* DE3 strain relative to *Rc*FDH^WT^ expression in the *E. coli* MC1061 strain, a lowered apparent % Mo saturation was expected. In turn, calculated formate oxidation *k*_cat_ values depicted herein are factored based upon the concentration of molybdenum, [Mo], as the amount of nonspecifically bound molybdate is small (14). Graphical box plots were prepared in OriginPro 2023b (OriginLab Corporation), depicting the mean, the median, and the interquartile range of 25 to 75% and the maximum of 1.5xIQR.

Time-dependent sulfuration was followed at selected timepoints by measuring the formate oxidation activity with excess formate and oxidized methyl viologen, with the above assay conditions employed, but without subsequent oxidative FMN reconstitution. Typical FDH sulfuration time-course experiments were performed in conjunction with a Shimadzu 1280 spectrophotometer housed in a Coy chamber (O_2_ < 20 ppm) with 300 *μ*l of approximately 30 *μ*M *Rc*FDH^ΔFdsC^ with 30 *μ*l of a sulfur source and 30 *μ*l of 120 mM Eu^II^DTPA (or of 100 mM Na_2_S_2_O_4_), which was incubated at 4 °C, in 75 mM potassium phosphate, pH 7.5 buffer and which contained or lacked 10 mM NaN_3_. Unless stated otherwise, Na_2_S_2_O_4_ was considered as both a sulfur source and a reductant. At selected timepoints, 3 *μ*l of the reaction mixture was diluted into 497 *μ*l of 75 mM potassium phosphate, 10 mM NaN_3_, pH 7.5 buffer, to dilute the amount of reductant and sulfur source to stop sulfuration. This mixture was then briefly concentrated at ambient temperature in an Eppendorf MiniSpin centrifuge to yield 80 to 100 μl of concentrated FDH stock that was approximately 0.7 to 1.2 *μ*M and was confirmed afterwards aerobically with the Shimadzu BioSpec-nano spectrophotometer. Formate oxidation activities were performed as described above with oxidized methyl viologen as an electron acceptor at ambient temperature, with 5 to 10 nM FDH present in the assay.

### EPR spectroscopy

Routine measurements to characterize the Mo^V^ oxidation state and the extent of sulfuration of the FDH bis-MGD cofactor of FDH^ΔFdsC^ were performed in Potsdam on a benchtop X-band Bruker Magnettech ESR5000 spectrometer (Bruker BioSpin) outfitted with a temperature controller module to allow for cooling of samples in a liquid nitrogen gas flow. Identical acquisition parameters were employed as described previously: temperature, 110 K; microwave frequency, 9.434 GHz, microwave power, 4 mW; modulation amplitude 2 G; modulation frequency, 100 kHz, sweep time, 90 s; *B*_0_, 340.00 mT; sweep, 50.00 mT (32). Spectra were also obtained in Berlin on a laboratory-built spectrometer equipped with a Bruker SHQ resonator with an ESR 910 helium flow cryostat with an ITC503 temperature controller (Oxford Instruments). Instrument components of the laboratory-built spectrometer in Berlin included a ER041MR microwave bridge (Bruker), a SR810 lock-in amplifier (Stanford Research System), and a 53181 A microwave counter (Agilent Technologies). A Cu^II^-EDTA standard was used as a reference for spin quantitation of FDH samples, while routine magnetic field calibrations to compensate field offsets between the Hall probe and the sample position were performed by measuring a reference N@C_60_ sample at ambient temperature (72, 73). Acquisition parameters for spectra obtained on the laboratory-built instrument at 80 K were similar to the 110 K parameters mentioned above, except that the microwave frequency was 9.370 GHz. Following sulfuration, enzyme aliquots from anaerobically frozen stock solutions were made aerobic and were reduced either with 10 mM formate or 10 mM Na_2_S_2_O_4_ in 75 mM potassium phosphate, 10 mM NaN_3_, pH 7.5 buffer. Formate reduction of sulfurated *Rc*FDH^WT^ in the presence of azide results in a distinct Mo^V^ species, featuring sharpened ^1^H hyperfine coupling associated with the solvent-exchangeable ^1^H at the sulfido ligand (31, 32). However, formate reduction in the composite mixtures of sulfurated and nonsulfurated bis-MGD cofactor resulted in reduction of only the sulfurated cofactor; no apparent reduction of the nonsulfurated bis-MGD cofactor was observed, although the possibility of partial reduction of Fe-S clusters cannot be ruled out (data not shown). By comparison, reduction with Na_2_S_2_O_4_ to composite mixtures of sulfurated and nonsulfurated bis-MGD cofactor results in the generation of discrete Mo^V^ species, respectively. In addition to the Mo^V^ signal(s), *Rc*FDH poised in the presence of excess reductant exhibits two [2Fe-2S] clusters G7 and A5 that are usually visible at 110 K. Spectral simulations were performed using the EasySpin simulation package (74) (version 6.0.5) in Matlab (MathWorks) version R2023a, and reported uncertainties for the simulated parameters are provided for the sulfurated Mo^V^ species detected herein. Standard deviation for all the Mo^V^, G7, and A5 Fe-S cluster *g-*values determined were on the order of 1 ⋅ 10^−5^; respective SD for the G7 and A5 Fe-S cluster *g-*strain parameters were on the order of 1 ⋅ 10^−4^ and 1 ⋅ 10^−3^, respectively. For the ^95,97^Mo spectral simulation component, Euler angles in the “AFrame” convention of EasySpin were employed and are noted in the footnotes of Table S1.

### Cofactor analysis

Mo and Fe metal analyses were routinely performed on as-purified *Rc*FDH^WT^, *Rc*FDH^ΔFdsC^, and *Rc*FDH^Δbis-MGD(WT)^ using a PerkinElmer Life Sciences Optima 2100DV inductively coupled plasma optical emission spectrometer as described previously (16, 68). Typical % Mo and % Fe saturations per protomer were 54.3 ± 2.3% and 54.3 ± 1.6% for *Rc*FDH^WT^ and 40.0 ± 1.2% and 53.8 ± 0.8% for *Rc*FDH^ΔFdsC^. Previously, the % Mo content in *Rc*FDH^ΔFdsC^ was lowered (75) relative to the quantity reported here; the increased % Mo content reported here reflects confirmed co-expression of FdsD during expression of *Rc*FDH^ΔFdsC^ in the *E. coli* BW25113 *ΔfdhD* DE3 strain. The % Fe saturation of *Rc*FDH^Δbis-MGD(WT)^ was 53.2 ± 1.2%, which represents the same enzyme stock reported previously (16). The bis-MGD cofactor bound in *Rc*FDH^WT^ and *Rc*FDH^ΔFdsC^ was detected fluorometrically as described previously, with minor deviations to the described procedure (68). Routine triplicate measurements of 200 *μ*l of 20 *μ*M FDH were analyzed. Relative activity per MGD plots represent data recordings comprising Form A-GMP quantitative measurements carried out on the same day, including identical treatment and handling of samples, as described previously for the enzyme trimethylamine-*N*-oxide reductase (59). Diluted stock FDH concentrations prior to the analysis were quantified with a Shimadzu BioSpec-nano spectrophotometer.

## Data availability

All data are contained within the manuscript. Raw experimental data can be provided from the authors upon reasonable request.

## Supporting information

This article contains supporting information. The supporting information includes cyanolysis quantitation, relative formate oxidation based upon quantified FormA-GMP, sulfide quantitation from dithionite and bisulfite-reduced samples, and supporting UV-Vis and EPR spectra (18, 32, 42, 43, 44).

## Conflicts of interest

The authors declare that they have no conflicts of interest with the contents of this article.
