# Supplementary material for: In vitro sulfuration of Rhodobacter capsulatus formate dehydrogenase
Source: J Biol Chem. 2025 Apr 15;301(6):108511. doi: 10.1016/j.jbc.2025.108511 (PMC12140954; doi:10.1016/j.jbc.2025.108511)
Supplement: Supporting information [file mmc1.docx]

**Supporting Information**

***In vitro* sulfuration of *Rhodobacter capsulatus* formate dehydrogenase**

Benjamin R. Duffus*^,a^, Benedict J. Elvers^b^, Christian Teutloff^c^, Carola Schulzke^b^, and Silke Leimkühler^a^

^a^Institute of Biochemistry and Biology, Department of Molecular Enzymology, University of Potsdam, 14476 Potsdam, Germany

^b^Institute of Biochemistry, Department of Bioinorganic Chemistry, University of Greifswald, 17489 Greifswald, Germany

^c^Institute of Experimental Physics, EPR Spectroscopy of Biological Systems, Freie Universität–Berlin, 14195 Berlin, Germany

**Table of Contents**

**Supporting Information Figures and Tables**

**Figure S1** Cyanolysis of *Rc*FDH^WT^ and respective *in vitro* sulfuration. 2

**Figure S2** UV-Vis reduction spectra of sulfurated *Rc*FDH^∆FdsC^ upon 3

treatment with formate.

**Figure S3** EPR spectral simulation of dithionite-reduced FDH^∆FdsC^, following 4

*in vitro* sulfuration in the presence of Eu^II^DTPA and Na_2_S.

**Figure S4** Reference *Rc*FDH EPR paramagnetic signals associated with a 5

bis-MGD cofactor with a sulfido or an oxo ligand.

**Figure S5** EPR spectral simulation of Na_2_S_2_O_4_-reduced FDH^∆FdsC^, 6

immediately frozen from the as-purified state without

incubation.

**Figure S6** Zoomed-in plot of EPR spectra depicting formate-reduced, sulfurated 7

FDH^∆FdsC^ and presulfurated FDH^WT^, with an emphasis on the

^1^H hyperfine present in the Mo^V^ signals present.

**Figure S7** UV-Visible spectral comparison of formate reduction of *in vitro* 8

sulfurated *Rc*FDH^∆FdsC^ samples.

**Figure S8** Relative formate oxidation of sulfurated *Rc*FDH^∆FdsC^, following *in vitro* 9

sulfuration with dithionite, using oxidized methyl viologen as an

electron acceptor.

**Figure S9** Reduction of *Rc*FDH^∆FdsC^ in the presence of Na_2_S_2_O_4_. 10

**Figure S10** Characterization of Mo^V^ species of *Rc*FDH^∆FdsC^ upon sulfur-free 11

reduction in the presence of bisulfite.

**Figure S11** Sulfide quantitation of *in vitro* sulfuration mixtures containing either 12

sodium dithionite or sodium bisulfite in the presence of Eu^II^DTPA

and FDH, using the sulfide-binding, fluoresecent reagent

4-chloro-7-nitrobenzofurazan

**Figure S12** CW X-band EPR spectral simulation of formate-reduced, 13

azide-inhibited FDH^∆FdsC^ following *in vitro* reconstitution with

Na_2_^33^S.

**Table S1** Simulation parameters of *R. capsulatus* FDH paramagnetic 14

redox centers presented in context to *in vitro* sulfuration of FDH^∆FdsC^

**Supporting Information References** 15


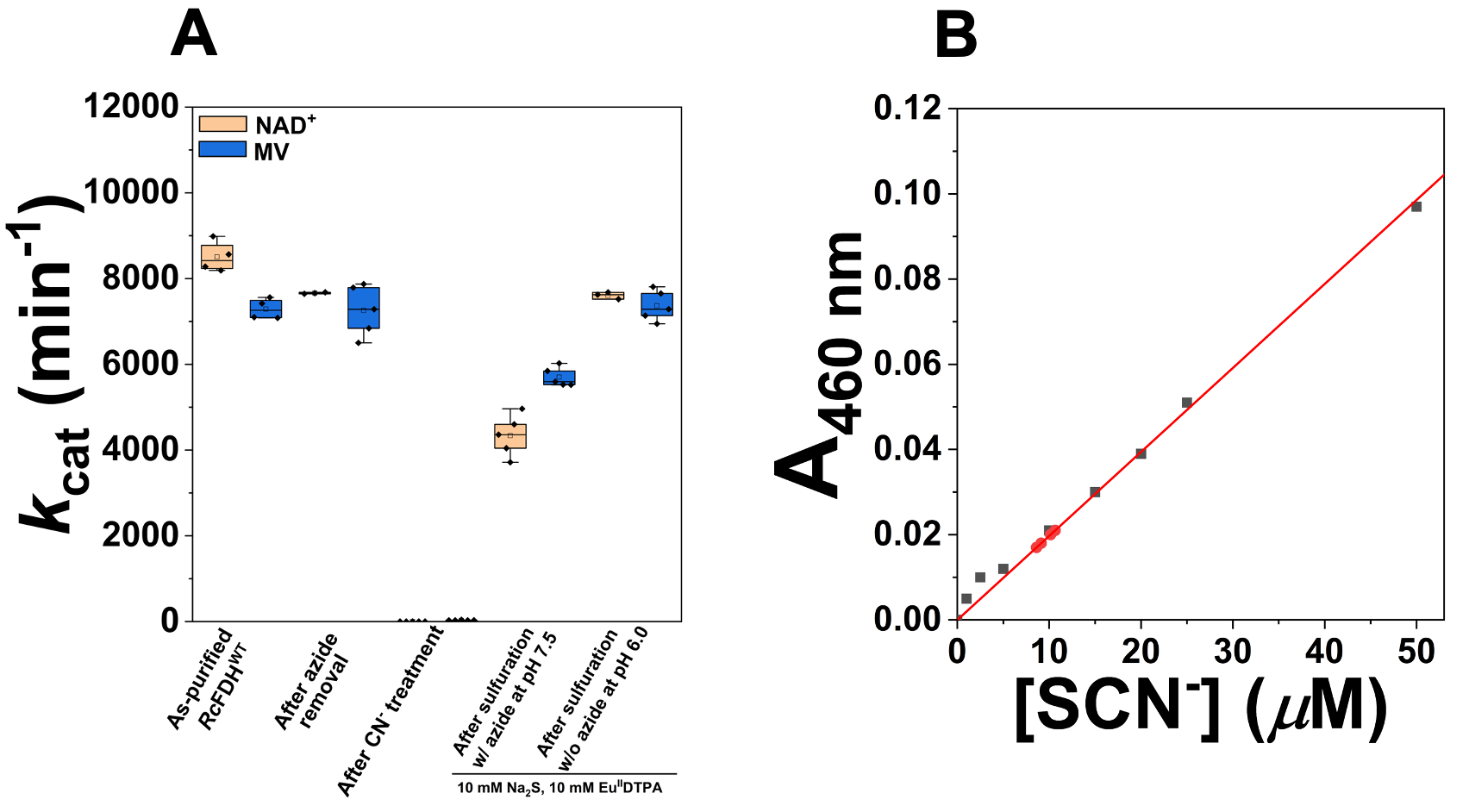


**Figure S1** Cyanolysis of *Rc*FDH^WT^ and respective *in vitro* sulfuration. Panel **A** depicts formate oxidation activities across handling of *Rc*FDH^WT^ upon cyanolysis and resulfuration of the identical *Rc*FDH^WT^ stock using either NAD^+^ or oxidized methyl viologen as electron acceptors. The resulfurated sample activity at pH 7.5 in the presence of 10 mM NaN_3_ represents the activity reported in **Figure 2A**. Please see the experimental methods section for more information regarding the formate oxidation activity assay applied here. Panel **B** depicts quantitation of thiocyanate derived from cyanolysis of *Rc*FDH^WT^ shown in Panel **A**. Black squares depict known analytical quantities of thiocyanate dissolved in 75 mM potassium phosphate, pH 7.5 that were added 1:1 to Sörbo’s reagent. Red circles represent the flow-through associated with treatment of 30 *µ*M *Rc*FDH^WT^ with 1 mM KCN in 75 mM potassium phosphate, pH 7.5 after incubation for 30 minutes at 10 °C and concentration of the enzyme. The averaged [SCN^‑^] in the flow-through was determined to be 9.6 ± 0.9 *µ*M. The % Mo saturation is 42.1 %, yielding an estimated [Mo] concentration of 12.6 *µ*M.


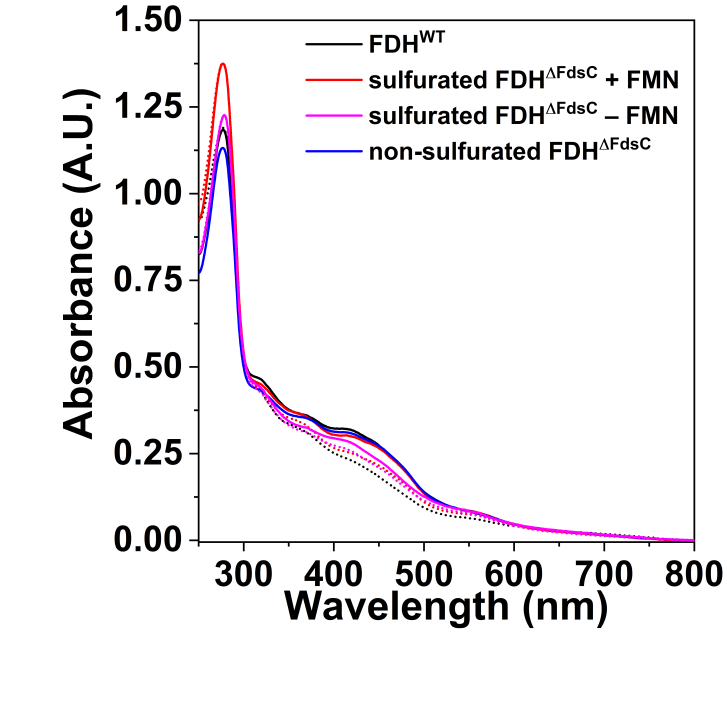


**Figure S2** UV-Vis reduction spectra of sulfurated *Rc*FDH^∆FdsC^ upon treatment with formate. Spectra represent the same plot depicted in **Figure 2B**, only zoomed out to encompass the 280 nm absorbance. Solid lines depict FDH samples in the as-isolated or as-obtained state following *in vitro* sulfuration, while the dotted lines depict respective spectra reduced with 5 mM sodium formate. For spectral comparison, reduction of sulfurated *Rc*FDH^WT^ with formate and the spectrum of as-isolated, non-sulfurated *Rc*FDH^∆FdsC^ are depicted as reference spectra, with respective spectra normalized relative to the FMN-associated and Fe-S cluster absorbances at 478 nm and 550 nm.

**
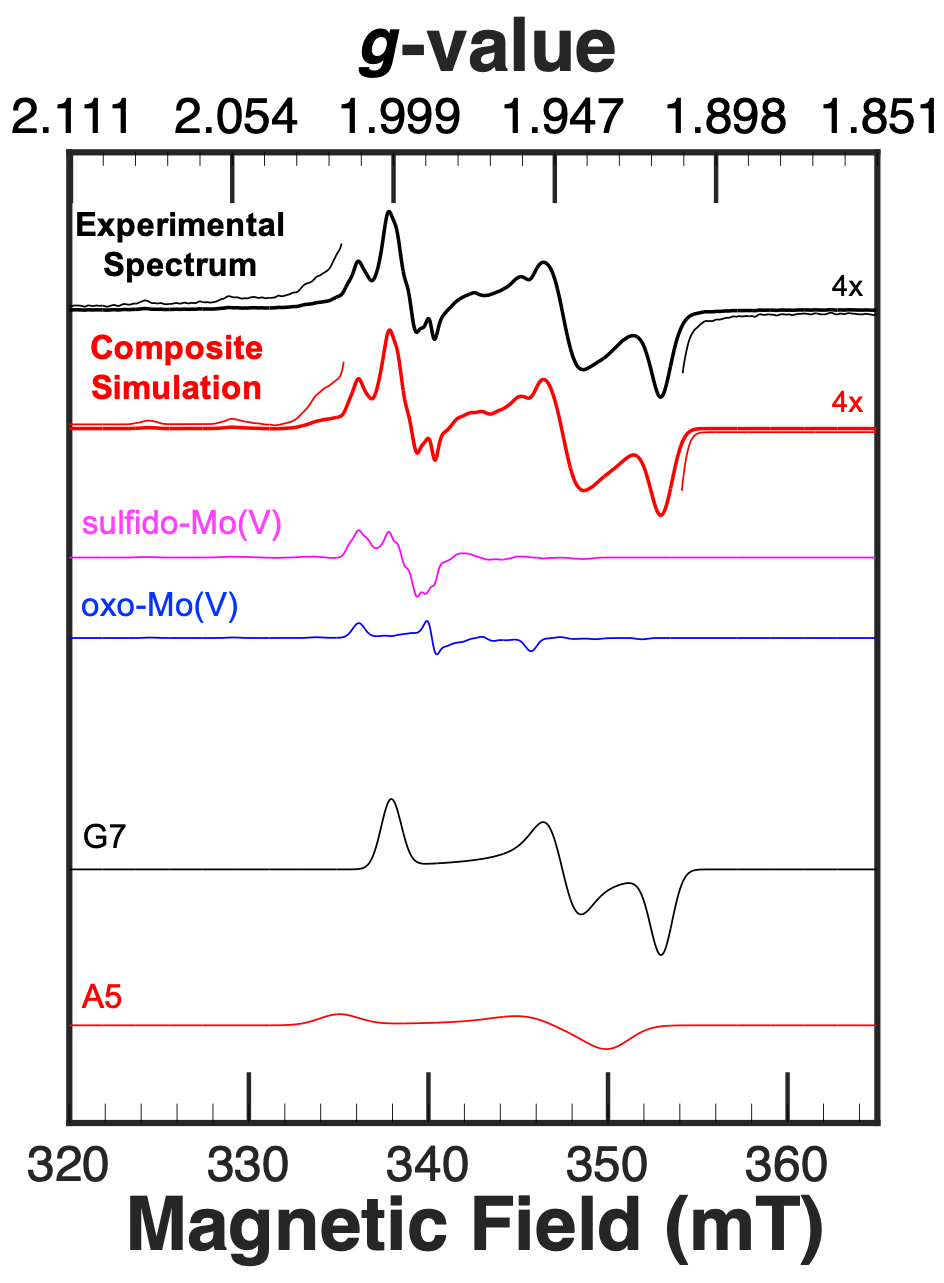
**

**Figure S3** EPR spectral simulation of dithionite-reduced FDH^∆FdsC^, following *in vitro* sulfuration in the presence of Eu^II^DTPA and Na_2_S. The experimental spectrum corresponds to the sulfurated FDH^∆FdsC^ spectrum depicted in **Figure 2C** (bottom spectrum), measured at 110 K at 4 mW microwave power, 2 G modulation amplitude, and 100 kHz modulation frequency. Composite simulation constitutes a mixture of sulfido and oxo Mo^V^, with additional spectral components corresponding to the G7 and A5 [2Fe-2S] clusters. Respective Mo^V^ simulation components include their respective ^95,97^Mo hyperfine components. Simulation weights for the sulfido-Mo^V^, oxo-Mo^V^ and G7 and A5 [2Fe-2S] cluster paramagnetic species were 5.8%, 5.7%, 66.4%, and 22.1%, respectively. A reference simulation for the purely oxo Mo^V^ species accompanying reduction of FDH^∆FdsC^ with Na_2_S_2_O_4_ is found in **Figure S4** and a corresponding simulation is found in **Figure S5**. For simulation parameters for the identified paramagnetic species, see **Table S1**.


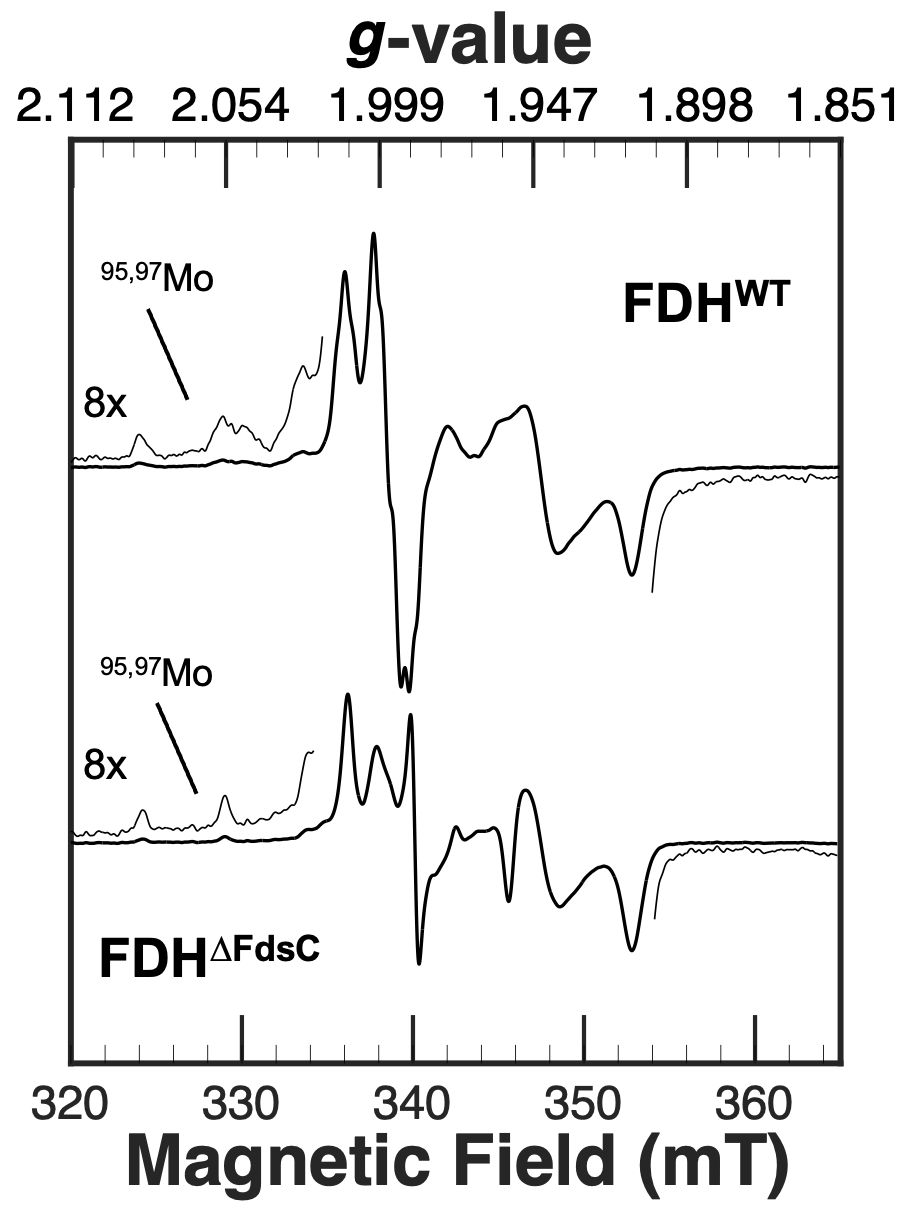


**Figure S4** Reference *Rc*FDH EPR paramagnetic signals associated with a bis-MGD cofactor with a sulfido or an oxo ligand. Plot depicts *Rc*FDH^WT^ (top) and RcFDH^∆FdsC^ (bottom) upon reduction with 10 mM Na_2_S_2_O_4_. Respective spectra represent an overlay of the G7 and A5 [2Fe-2S] clusters, and the associated paramagnetic Mo^V^ species. EPR samples were prepared in 75 mM potassium phosphate, 10 mM NaN_3_, pH 7.5 buffer. Spectra were obtained at 110 K, at 9.45 GHz microwave frequency and at 4 mW microwave power with a 2 G modulation amplitude and a 100 kHz modulation frequency. Spectra are normalized relative to the G7 [2Fe-2S] cluster present. The ^95,97^Mo hyperfine present in the baseline is additionally depicted 6x. For more information regarding the *g-*tensor parameters for the individual paramagnetic species identified, see **Table S1**.


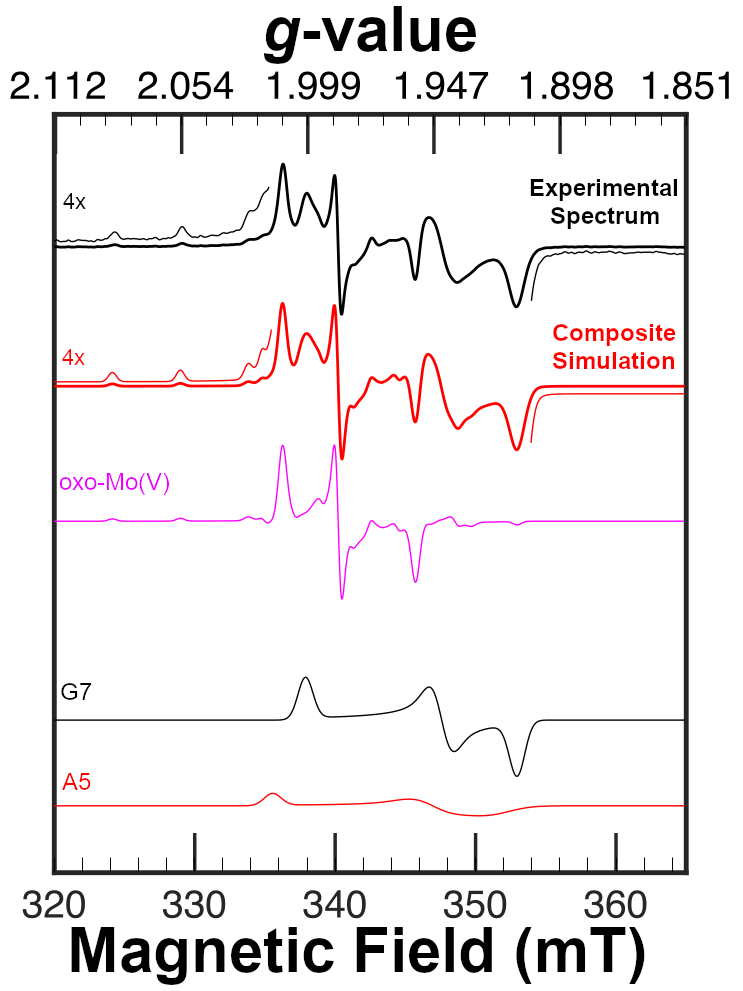


**Figure S5** EPR spectral simulation of Na_2_S_2_O_4_-reduced FDH^∆FdsC^, immediately frozen from the as-purified state without incubation. The experimental spectrum corresponds to the sulfurated FDH^∆FdsC^ spectrum depicted in **Figure S4** (bottom spectrum), measured at 110 K at 4 mW microwave power, 2 G modulation amplitude, and 100 kHz modulation frequency. Associated ^95,97^Mo hyperfine in the baseline is depicted 4x. Composite simulation constitutes an oxo Mo^V^ species with associated ^95,97^Mo hyperfine, with additional spectral components corresponding to the A5 and G7 [2Fe-2S] clusters. Simulation weights for the oxo-Mo^V^, the G7, and the A5 [2Fe-2S] clusters were 31.8%, 50.2%, and 18.0%, respectively. For simulation parameters for the identified paramagnetic species, see **Table S1**.


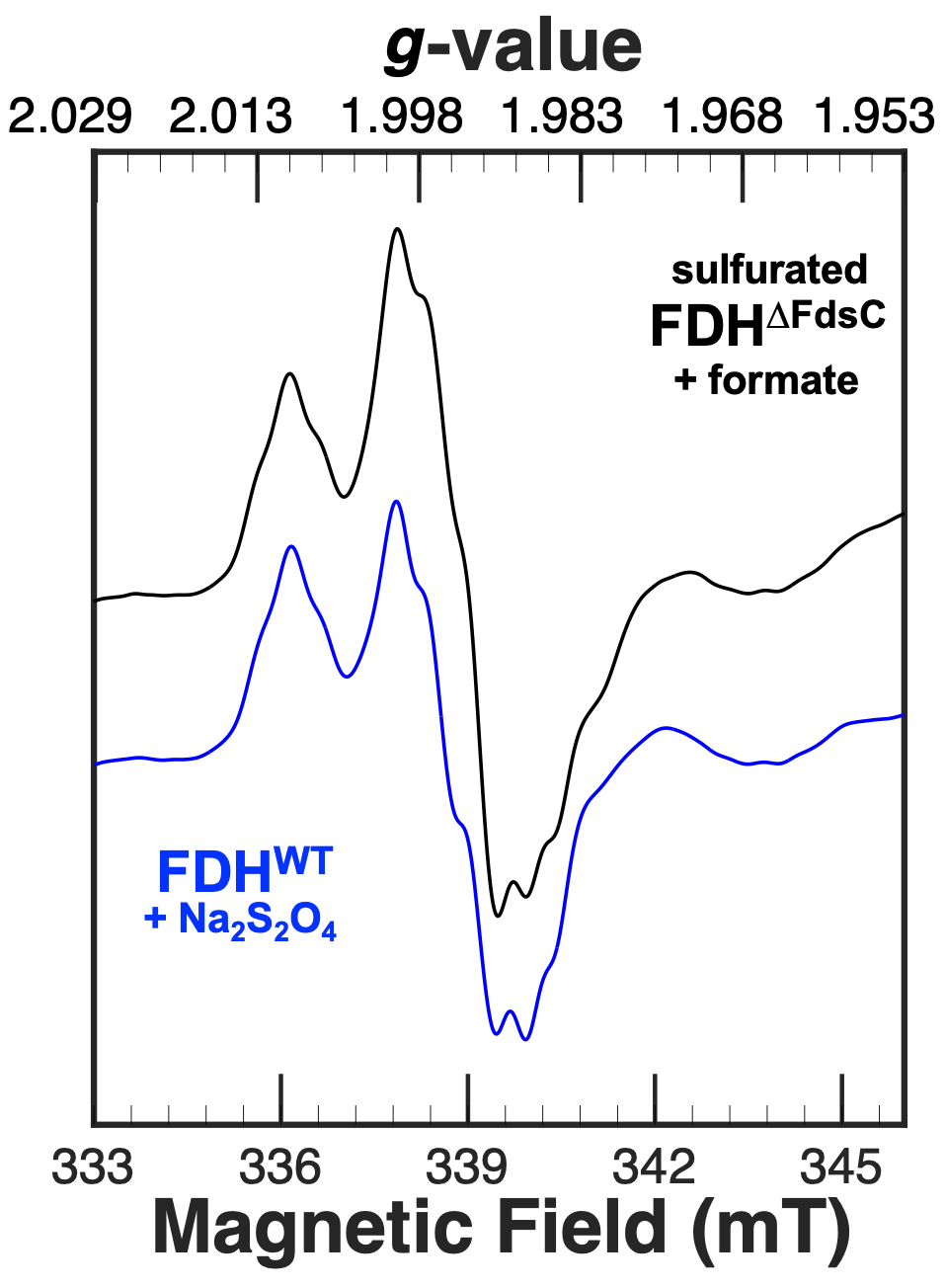


**Figure S6** Zoomed-in plot of EPR spectra depicting formate-reduced sulfurated FDH^∆FdsC^ and presulfurated FDH^WT^, with an emphasis on the ^1^H hyperfine present in the Mo^V^ signals present. Plot represents the formate-reduced samples depicted in **Figure 2C** plotted with a narrower magnetic field range. EPR samples were prepared in 75 mM potassium phosphate, 10 mM NaN_3_, pH 7.5 buffer. Spectra were obtained at 110 K, at 9.45 GHz microwave frequency and at 4 mW microwave power with a 2 G modulation amplitude and a 100 kHz modulation frequency. The FDH^WT^ spectrum (blue trace) is normalized relative to the Mo^V^ signal present in the sulfurated FDH^∆FdsC^ spectrum (black trace). For additional information regarding the *g-*tensor parameters for the paramagnetic species identified, see **Table S1**.


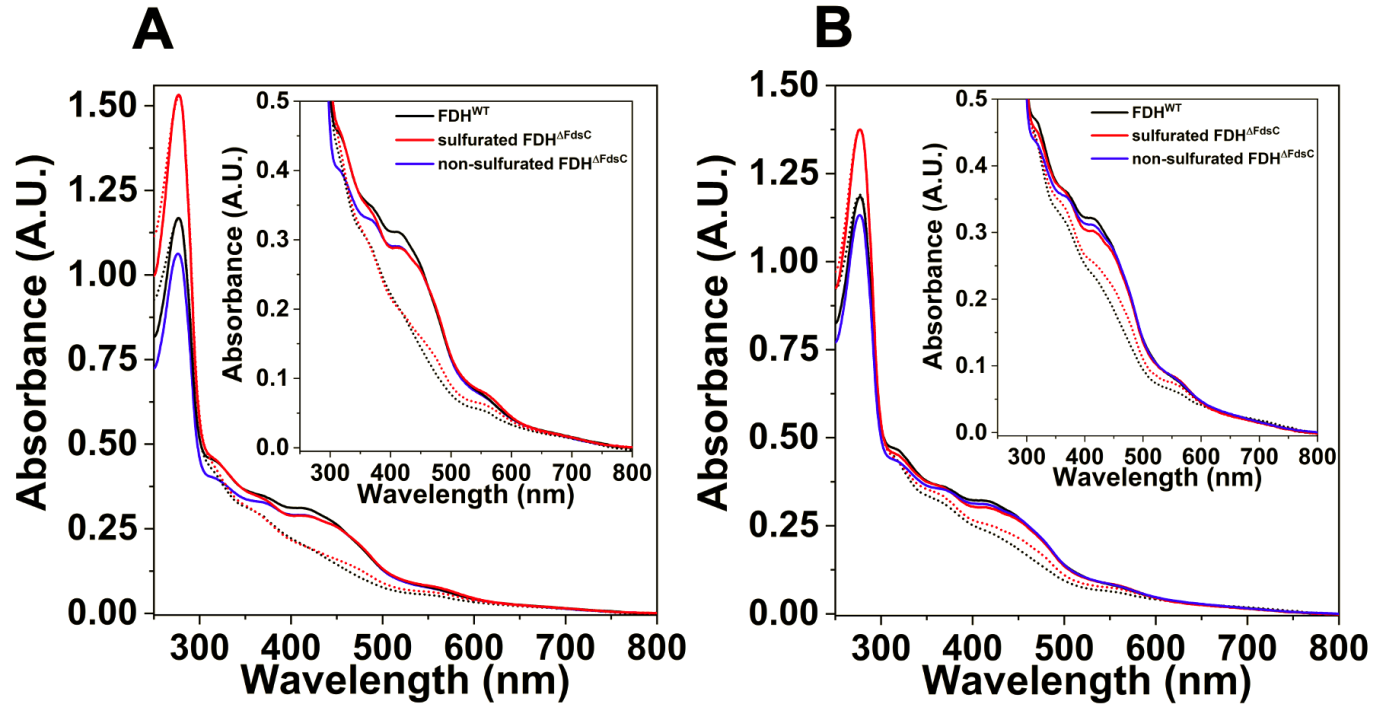


**Figure S7** UV-Visible spectral comparison of formate reduction of *in vitro* sulfurated *Rc*FDH^∆FdsC^ samples. Solid lines depict FDH in the as-isolated or as-obtained state, while the dotted lines represent spectra following reduction with formate. Panel **A** depicts *Rc*FDH^∆FdsC^ sulfurated with 5 mM Na_2_S_2_O_4_ in the absence of azide, whose relative formate oxidation activity is depicted in **Figure 4** in the main text. Panel **B** depicts *Rc*FDH^∆FdsC^ sulfurated with 10 mM Na_2_S in the presence of 10 mM Eu^II^DTPA and in the presence of azide, whose activity is depicted in **Figure 2A**. UV-Visible spectra are normalized relative to the FMN feature at 478 nm and the Fe-S cluster related feature at 550 nm in the as-isolated or as-obtained state. For reference, respective UV-Visible spectra for *Rc*FDH^WT^ and non-sulfurated *Rc*FDH^∆FdsC^ are also depicted.


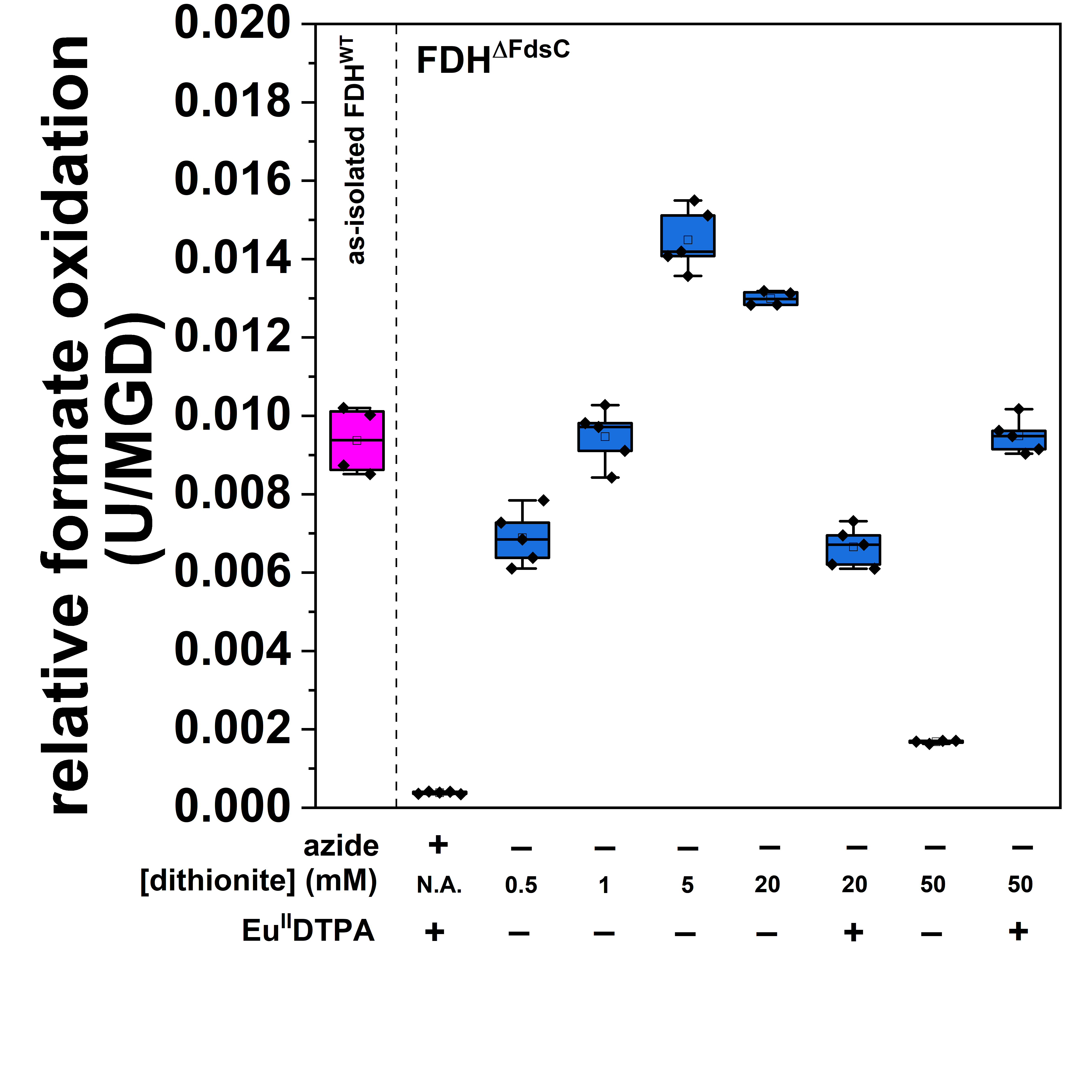


**Figure S8** Relative formate oxidation of sulfurated *Rc*FDH^∆FdsC^, following *in vitro* sulfuration with dithionite, using oxidized methyl viologen as an electron acceptor. The relative formate oxidation activity represents the specific formate oxidation activity, divided by the detected amount of FormA-GMP detected in the sample per mg of FDH. The blue bars depict *Rc*FDH^∆FdsC^ that underwent sulfuration with Na_2_S_2_O_4_ as a sulfur source and reductant, in the absence of 10 mM NaN_3_. For a select number of samples, 10 mM Eu^II^DTPA was also added. The magenta bar represents formate oxidation activity of pre-sulfurated *Rc*FDH^WT^ in the as-purified state. All depicted *Rc*FDH^∆FdsC^ sulfurations reflect sample that underwent oxidative FMN reconstitution after sulfuration; samples were isolated in 75 mM potassium phosphate, 10 mM NaN_3_, pH 7.5 buffer. The approximate concentration of the sulfur source is depicted, although it should be noted that the dithionite used in the assay is maximally 85% pure. N.A. = no Na_2_S_2_O_4_ addition.


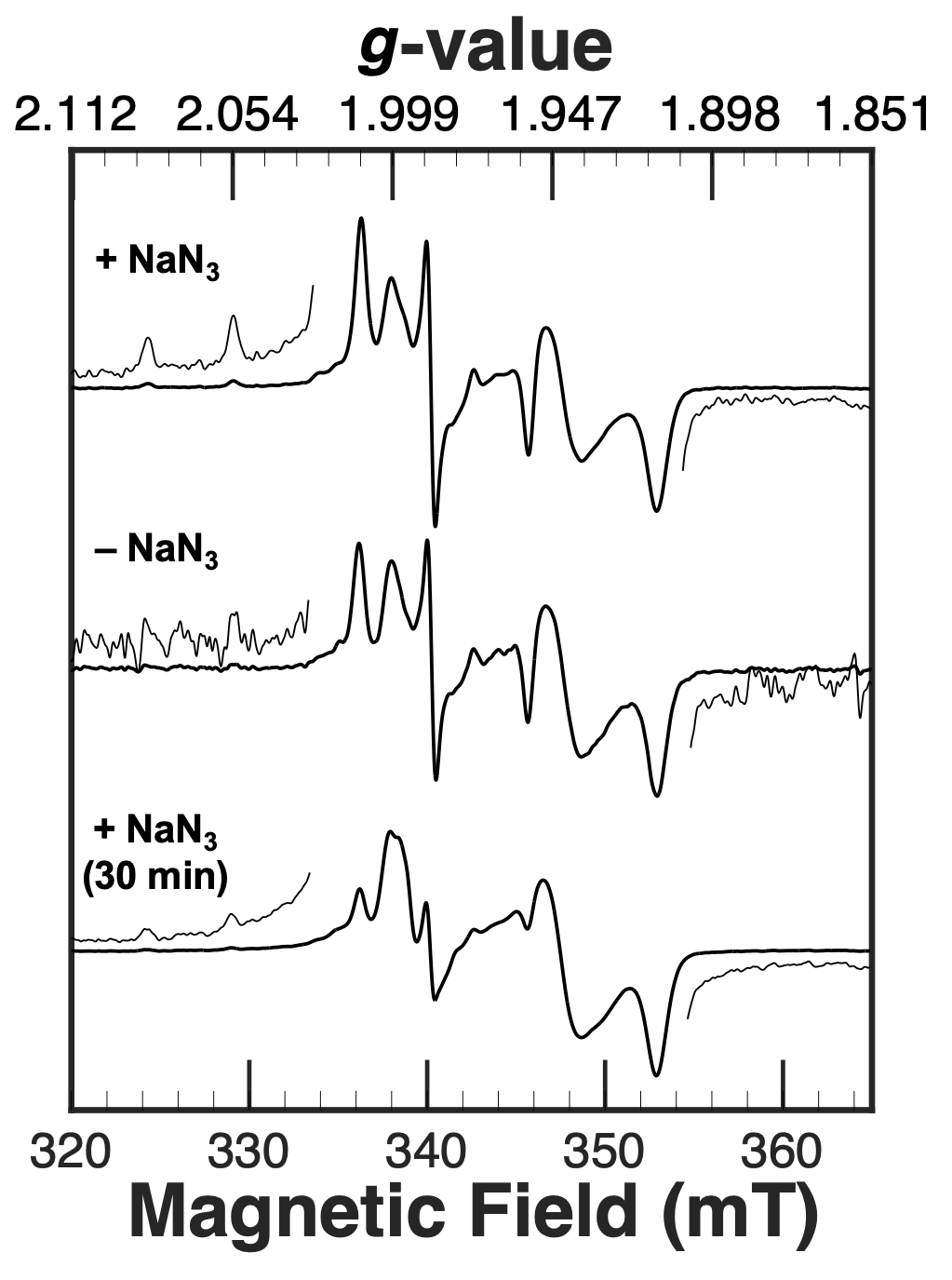


**Figure S9** Reduction of *Rc*FDH^∆FdsC^ in the presence of Na_2_S_2_O_4_. Samples represent *Rc*FDH^∆FdsC^ following aerobic treatment with 10 mM Na_2_S_2_O_4_ in the presence or absence of 10 mM NaN_3_. Samples were either promptly (~20 s) frozen, or were incubated for 30 minutes at 4 °C prior to sample freezing. Spectra were obtained at 110 K with a 9.45 GHz microwave frequency at 4 mW microwave power, 2 G modulation amplitude, and 100 kHz modulation frequency. Spectra were normalized relative to the reduced G7 [2Fe-2S] cluster species. To aid in the depiction of the ^95,97^Mo hyperfine features, the signal baseline is depicted 8x. For associated simulation parameters for the Mo^V^ signal(s), see **Table S1** and **Figure S5**.


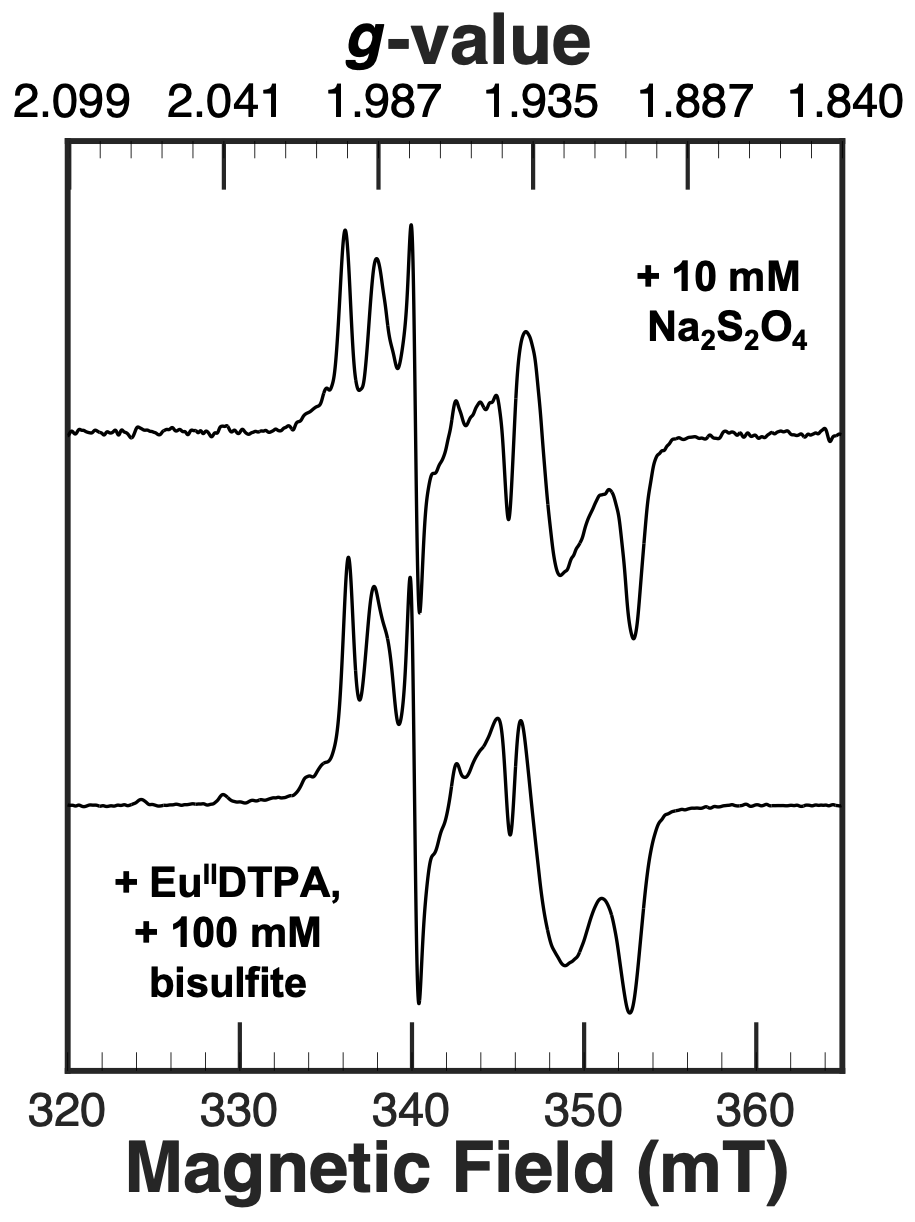


**Figure S10** Characterization of Mo^V^ species of *Rc*FDH^∆FdsC^ upon reduction in the presence of bisulfite. The top spectrum represents *Rc*FDH^∆FdsC^ treated with 10 mM Na_2_S_2_O_4_, that was promptly (~20 s) frozen in liquid N_2_. The bottom spectrum also represents *Rc*FDH^∆FdsC^ that was treated with 750 *µ*M Eu^II^DTPA and 100 mM NaHSO_3_. Samples were handled anaerobically, and were prepared in 75 mM potassium phosphate, 10 mM NaN_3_, pH 7.5 buffer. The [FDH] is 300 *µ*M. Spectra were recorded at 9.43 GHz microwave frequency, 110 K, 4 mW microwave power, 2 G modulation amplitude, and 100 kHz modulation frequency.


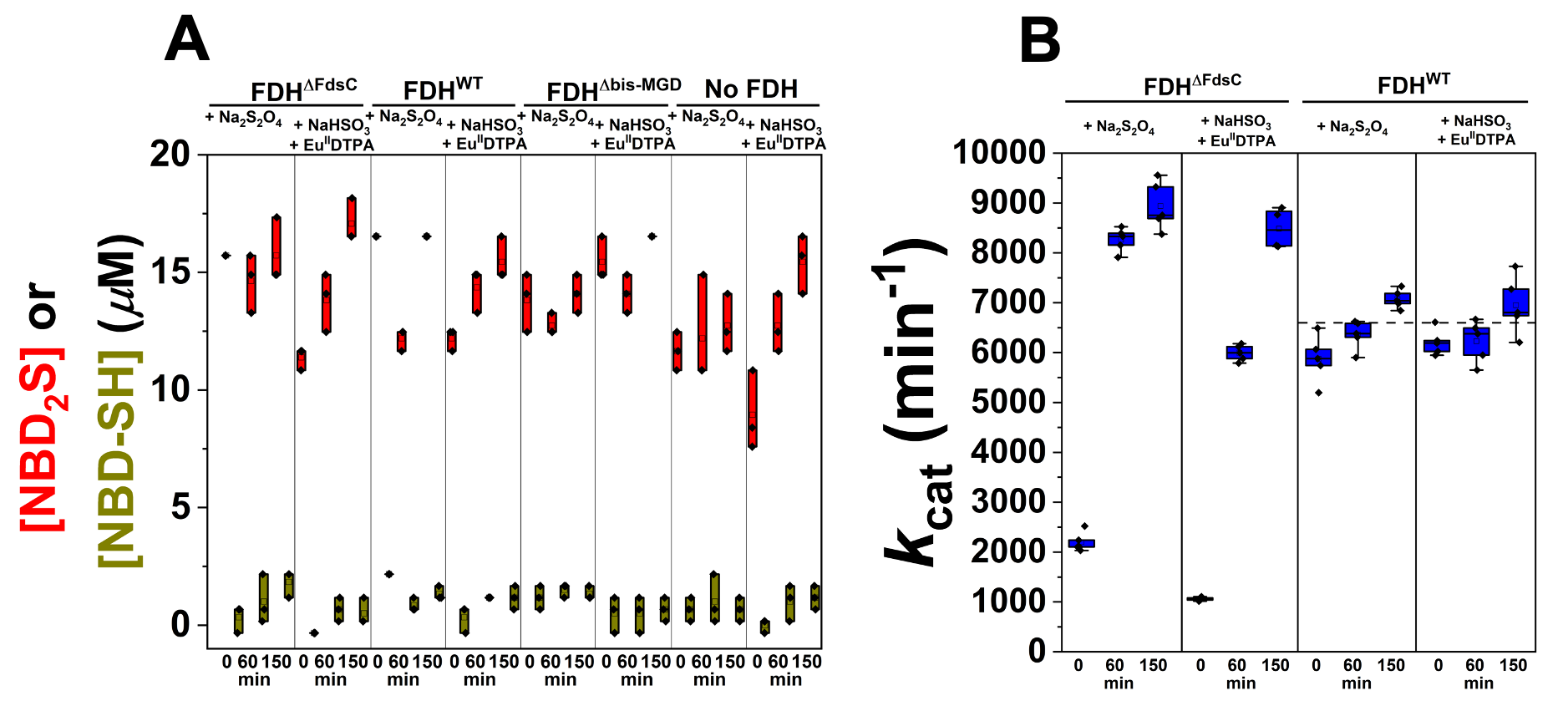


**Figure S11** Sulfide quantitation of *in vitro* sulfuration mixtures containing either sodium dithionite (Na_2_S_2_O_4_) or sodium bisulfite in the presence of Eu^II^DTPA and FDH, using the sulfide-binding, fluorescent reagent 4-chloro-7-nitrobenzofurazan (NBD-Cl). Panel **A** depicts assay incubations that were performed in 75 mM potassium phosphate, pH 7.5 in the presence of 10 mM Na_2_S_2_O_4_ or 10 mM NaHSO_3_ with 10 mM Eu^II^DTPA, and where applicable, 32 *µ*M *Rc*FDH^∆FdsC^, 24 *µ*M *Rc*FDH^WT^, or 22 *µ*M *Rc*FDH^∆bis-MGD(WT)^. The added final concentration of NBD-Cl to reaction mixtures or to flow-through after removing FDH is 80 *µ*M. Panel **B** depicts respective formate oxidation activity on the retained FDH after removal of the flow-through and buffer exchange into 75 mM potassium phosphate, pH 7.5, performed in the presence of oxidized methyl viologen as an electron acceptor. The time required to thoroughly exchange the excess sulfur source out of the FDH sample was approximately 30 minutes. The dashed line in the FDH^WT^ measurements in Panel **B** represents the apparent formate oxidation activity prior to *in vitro* sulfuration.


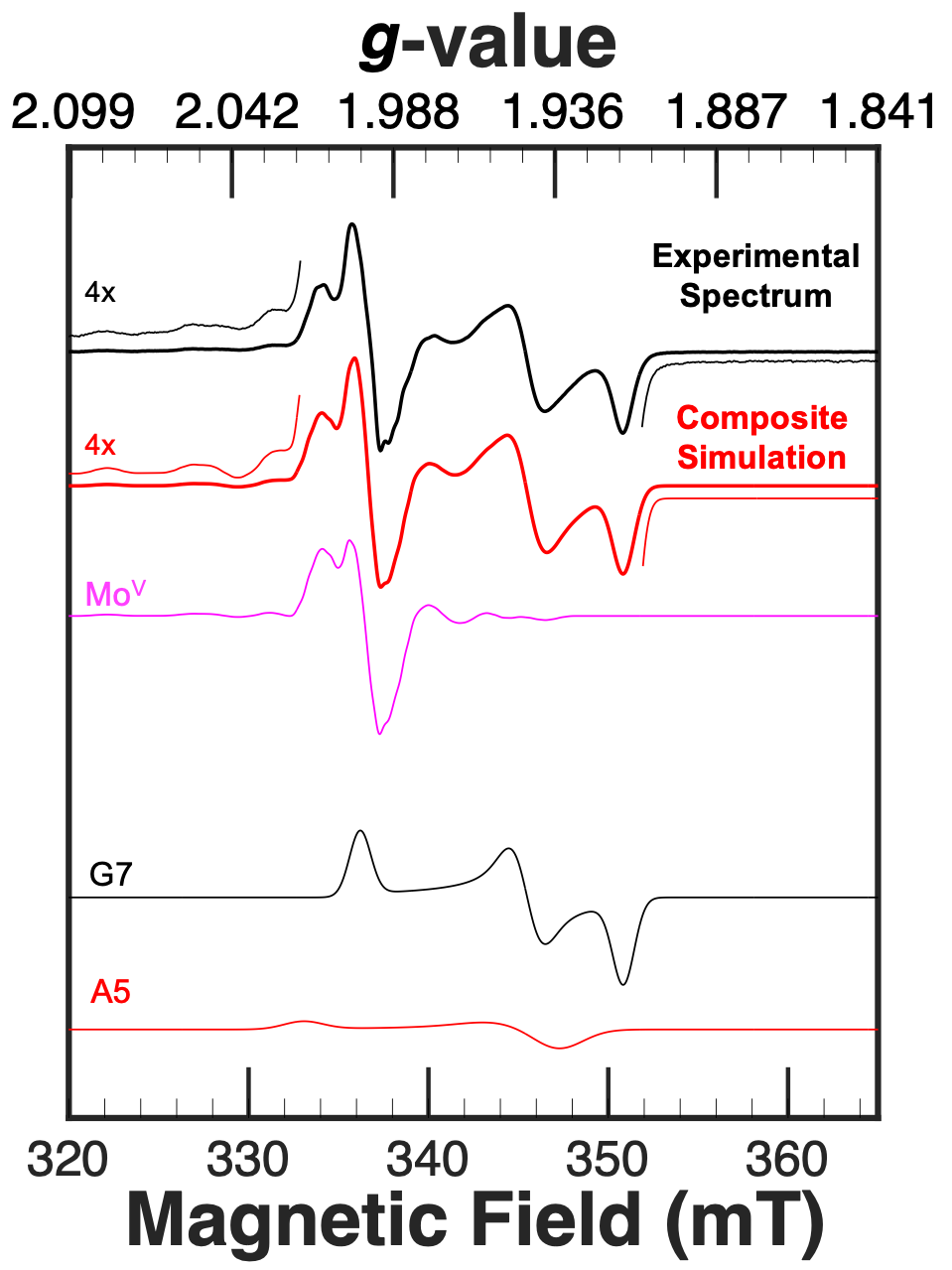


**Figure S12** CW X-band EPR spectral simulation of formate-reduced, azide-inhibited FDH^∆FdsC^ following *in vitro* reconstitution with Na_2_^33^S. Experimental spectrum represents the isotopically labeled spectrum depicted in **Figure 7** that was measured at 80 K at 9.38 GHz microwave frequency, 4 mW microwave power, 2 G modulation amplitude, and 100 kHz modulation frequency. The composite simulation represents the spectral components visible at 80 K, that includes the G7 and A5 [2Fe-2S] clusters in addition to the Mo^V^ species. Spectral components are overlaid below in respective colors. Spectral parameters for the Mo^V^ species can be found in **Table S1**.

**Table S1** Simulation parameters of *R. capsulatus* FDH paramagnetic redox centers presented in context to in vitro sulfuration of FDH^∆FdsC^

| Signal type: | Active Site Ligand | *g-*tensor values | *g*_av_ | *g*_aniso_ | *g*_rhomb_ | *g-*strain values  (x10^-3^) | H-strain  values  (MHz) | ^95,97^Mo  hyperfine parameters (MHz) | ^1^H hyperfine parameters  (MHz) | ^33^S hyperfine  parameters (MHz) | Reference |
| --- | --- | --- | --- | --- | --- | --- | --- | --- | --- | --- | --- |
| G7 | N.A. | 2.002, 1.946, 1.917 | 1.956 | 0.085 | 0.660 | 6.82, 12.6, 8.10 |  |  |  | N.A. | (1) |
| A5 | N.A. | 2.021, 1.952, 1.933 | 1.969 | 0.088 | 0.793 | 19.1, 13.1, 14.8 |  |  |  | N.A. | (1) |
| (pre-sulfurated)  FDH^WT^ Mo^V^ + azide*^a^* | Cys | 2.012, 1.997, 1.989 | 1.999 | 0.023 | 0.638 |  | 14.0 ± 0.4, 10.8 ± 0.3, 11.8 ± 0.4 | 135.0 ± 0.9, 84.0 ± 0.7, 44.0 ± 1.3 | #1: 12.9 ± 0.5, 11.8 ± 0.4, 10.5 ± 0.3  #2: 15.3 ± 0.5, 18.7 ± 0.3, 16.3 ± 0.3 | N.A. | ((1), this work) |
| *in vitro* natural abundance sulfurated  Mo^V^ + azide*^a^* | Cys | 2.012, 1.997,  1.989 | 1.999 | 0.023 | 0.638 |  | 14.0 ± 1.0,  10.8 ± 0.6, 11.8 ± 0.5 | 135.0 ±1.0, 84.0 ± 0.8, 44.0 ± 2.3 | #1: 12.9 ± 1.4, 11.8 ± 0.5, 10.5 ± 0.3  #2: 15.3 ± 1.5, 18.7 ± 0.4, 16.3 ± 0.4 | N.A. | this work |
| *in vitro* ^33^S-sulfurated  Mo^V^ + azide*^a^* | Cys | 2.012, 1.997, 1.989 | 1.999 | 0.023 | 0.638 |  | 14.0 ± 3.2,  10.8 ± 4.1  11.8 ± 5.1 | 135.0 ± 3.3, 84.0 ± 2.3, 44.0 ± 19.6 | #1: 12.9 ± 6.8, 11.8 ± 1.3, 10.5 ± 4.1  #2: 15.3 ± 5.7, 18.6 ± 1.1, 16.3 ± 2.6 | 11.1 ± 0.5, 4.2 ± 0.7, 10.2 ± 0.8 | this work |
| Na_2_S_2_O_4_-reduced Mo^V^ FDH^∆FdsC^ + azide*^b^* | Cys | 2.010, 1.986, 1.954 | 1.983 | 0.055 | 0.424 |  | 18.9 ± 0.4, 13.4 ± 0.1, 19.1 ± 0.1 | 138.3 ± 0.3, 41.2 ± 0.4, 73.7 ± 1.0 | N.A. | N.A. | this work |
| Eu^II^DTPA-reduced FDH^∆FdsC^ + azide + bisulfite*^b^* | Cys | 2.011, 1.987, 1.956 | 1.985 | 0.055 | 0.430 |  | 19.4 ± 0.2,  12.5 ± 0.1,  18.9 ± 0.1 | 138.3 ± 0.3, 45.1 ± 0.6, 60.6 ± 1.0 | N.A. | N.A. | this work |
| *M. formicicum*  FDH^WT^ Mo^V^ + azide | Cys | 2.020, 2.006, 1.997 | 2.008 | 0.023 | 0.609 |  |  |  | #1: 12.6, 15.4, 14.0  #2: 12.6, 15.4, 14.0 |  | (2) |
| *D. desulfuricans* FDH Mo^V^ | Sec | 2.019, 1.988, 1.963 | 1.990 | 0.056 | 0.554 |  |  |  |  |  | (3) |
| *P. aeringosa* FDH Mo^V^ #1 | Sec | 2.012, 1.985, 1.968 | 1.988 | 0.044 | 0.614 |  |  |  |  |  | (4) |
| *P. aeringosa* FDH Mo^V^ #2 | Sec | 1.996, 1.981, 1.941 | 1.973 | 0.055 | 0.273 |  |  |  |  |  | (4) |
| *E. coli* DMSOR S176A Mo^V^ | Ala | 2.018, 1.982, 1.961 | 1.987 | 0.056 | 0.643 |  |  |  |  |  | (5) |

N.A. not applicable

*^a^*The ^95,97^Mo simulation component had the AFrame Euler angles (α, β, γ in degrees) of (9, 9, 155).

*^b^*The ^95,97^Mo simulation component had the AFrame Euler angles (α, β, γ in degrees) of (56, 32, 120).

**Supporting Information References:**

1. Duffus, B. R., Gauglitz, M., Teutloff, C., and Leimkühler, S. (2024) Redox potentials elucidate the electron transfer pathway of NAD^+^-dependent formate dehydrogenases. *J. Inorg. Biochem.* **253**, 112487 10.1016/j.jinorgbio.2024.112487

2. Barber, M. J., May, H. D., and Ferry, J. G. (1986) Inactivation of Formate Dehydrogenase from *Methanobacterium formicicum* by Cyanide. *Biochemistry* **25**, 8150-8155 10.1021/bi00373a004

3. Costa, C., Teixeira, M., LeGall, J., Moura, J. J. G., and Moura, I. (1997) Formate dehydrogenase from *Desulfovibrio desulfuricans* ATCC 27774: Isolation and spectroscopic characterization of the active sites (heme, iron-sulfur centers and molybdenum). *J. Biol. Inorg. Chem.* **2**, 198-208 10.1007/s007750050125

4. Gadsby, P. M. A., Greenwood, C., Coddington, A., Thomson, A. J., and Godfrey, C. (1987) Purification and Properties of Formate Dehydrogenase from *Pseudomonas aeruginosa* - Electron-Paramagnetic-Resonance Studies on the Molybdenum Center. *Biochem. J.* **243**, 235-239 10.1042/bj2430235

5. Trieber, C. A., Rothery, R. A., and Weiner, J. H. (1996) Consequences of Removal of a Molybdenum Ligand (DmsA-Ser-176) of *Escherichia coli* Dimethyl Sulfoxide Reductase. *J. Biol. Chem.* **271**, 27339-27345 10.1074/jbc.271.44.27339
